# NOX5 mediates the crosstalk between tumor cells and cancer‐associated fibroblasts via regulating cytokine network

**DOI:** 10.1002/ctm2.472

**Published:** 2021-08-03

**Authors:** Jie Chen, Yan Wang, Weimin Zhang, Di Zhao, Lingyuan Zhang, Jing Zhang, Jiawen Fan, Qimin Zhan

**Affiliations:** ^1^ Key Laboratory of Carcinogenesis and Translational Research (Ministry of Education/Beijing) Laboratory of Molecular Oncology Peking University Cancer Hospital & Institute Beijing China; ^2^ Institute of Cancer Research Shenzhen Bay Laboratory Shenzhen China; ^3^ Research Unit of Molecular Cancer Research Chinese Academy of Medical Sciences Beijing China

**Keywords:** adipose‐derived mesenchymal stem cells, cancer‐associated fibroblasts, esophageal squamous cell carcinoma, normal fibroblasts, NOX5

## Abstract

Activation of cancer‐associated fibroblasts (CAFs) is a crucial feature for tumor malignancy. The reciprocal interplay between tumor cells and CAFs not only facilitates tumor progression and metastasis but also sustains the tumor‐promoting function of CAFs. Nevertheless, how tumor cells readily adapt to these functional CAFs is still unclear. NADPH oxidase 5 (NOX5) is a strong reactive oxygen species producer overexpressed in esophageal squamous cell carcinoma (ESCC) cells. In this study, we showed that NOX5‐positive ESCC cells induced normal fibroblasts (NFs) or adipose‐derived mesenchymal stem cells (MSCs) to express the marker of CAFs‐α smooth muscle actin. Moreover, these tumor cells reprogrammed the cytokine profile of the activated CAFs, which further stimulated NFs or MSCs to CAFs and induced lymphangiogenesis to facilitate ESCC malignancy. NOX5 activated intratumoral Src/nuclear factor‐κB signaling to stimulate secretion of tumor necrosis factor‐α (TNF‐α), interleukin‐1β (IL‐1β), and lactate from tumor cells. Subsequently, TNF‐α, IL‐1β, and lactate activated CAFs, and facilitated the secretion of IL‐6, IL‐7, IL‐8, CCL5, and transforming growth factor‐β1 from CAFs. These CAFs‐derived cytokines reciprocally induced the progression of NOX5‐positive ESCC cells. Our findings together indicate that NOX5 serves as the driving oncoprotein to provide a niche that is beneficial for tumor malignant progression.

## Graphical abstract

1. NOX5‐controlled signaling/cytokine loop mediates crosstalk between ESCC and stroma

2. NOX5 activates Src/NF‐κB signaling to facilitate secretion of TNF‐α, IL‐1β and lactate from ESCC cells, and these substances induce the activation of NFs and adipose‐derived MSCs into CAFs

3. IL‐6, IL‐7, IL‐8, CCL5, and TGF‐β1 secreted from activated‐CAFs reciprocally promote the malignant progression of NOX5‐positive ESCC cells.

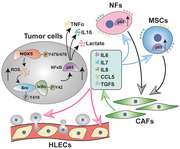

## 1 INTRODUCTION

The activation and functional characteristics of tumor cells and their surrounding microenvironments are regulated by tumor and the stroma interactions during tumor progression.^1^, ^2^, ^3^ Cancer‐associated fibroblasts (CAFs), an important cellular component of tumor microenvironment (TME), play profound roles in tumor pathogenesis, and correlate with significant clinical implications.^4^, ^5^, ^6^ CAFs include various origins of reprogrammed normal fibroblasts (NFs), which are raised by tumor cells to favor tumoral malignant phenotypes via secreting various cytokines and other factors.^7^, ^8^, ^9^, ^10^, ^11^, ^12^ Although comprehensive analyses of cytokine expression profiles in CAFs have identified in several cancer types,^13^, ^14^, ^15^ there have been no systematic comparison of cytokine profiles between CAFs and their origin NFs. It is not clear how CAFs are transited from NFs and what are the mechanism(s) by which this transition is controlled by tumor cells during tumor malignancy.

Human esophageal squamous cell carcinoma (ESCC) encompasses specific esophageal cancer subtype with unclear cellular and mutational heterogeneity, which lead to uncontrolled clinical outcomes and lack of effectively therapeutic targets in ESCC treatment.^16^ Our previous study demonstrated that the NADPH oxidase 5 (NOX5) level in ESCC tissue samples was tightly related to tumor malignant progression and the shorter survival of ESCC patients. It also showed that NOX5‐produced reactive oxygen species (ROS) activated several oncogenic signaling pathways, indicating that NOX5 may serve as a target for ESCC diagnosis and treatment.^17^ Tumor cells educate TME through activation of signaling pathways and secretion of various factors, and then TME reciprocally facilitates tumor malignancy.^3^, ^18^ Nevertheless, the functionality of NOX5 in mediating tumor/stroma crosstalk remains a huge puzzle. In the present research, we investigated the interaction between NOX5‐positive cancer cells and CAFs, and examined the underlying mechanisms.

## 2 MATERIALS AND METHODS

### 2.1 Information of antibodies and reagents

Information of antibodies and reagents is listed in Table S1.

### 2.2 Cell culture and transfection

**FIGURE 1:**
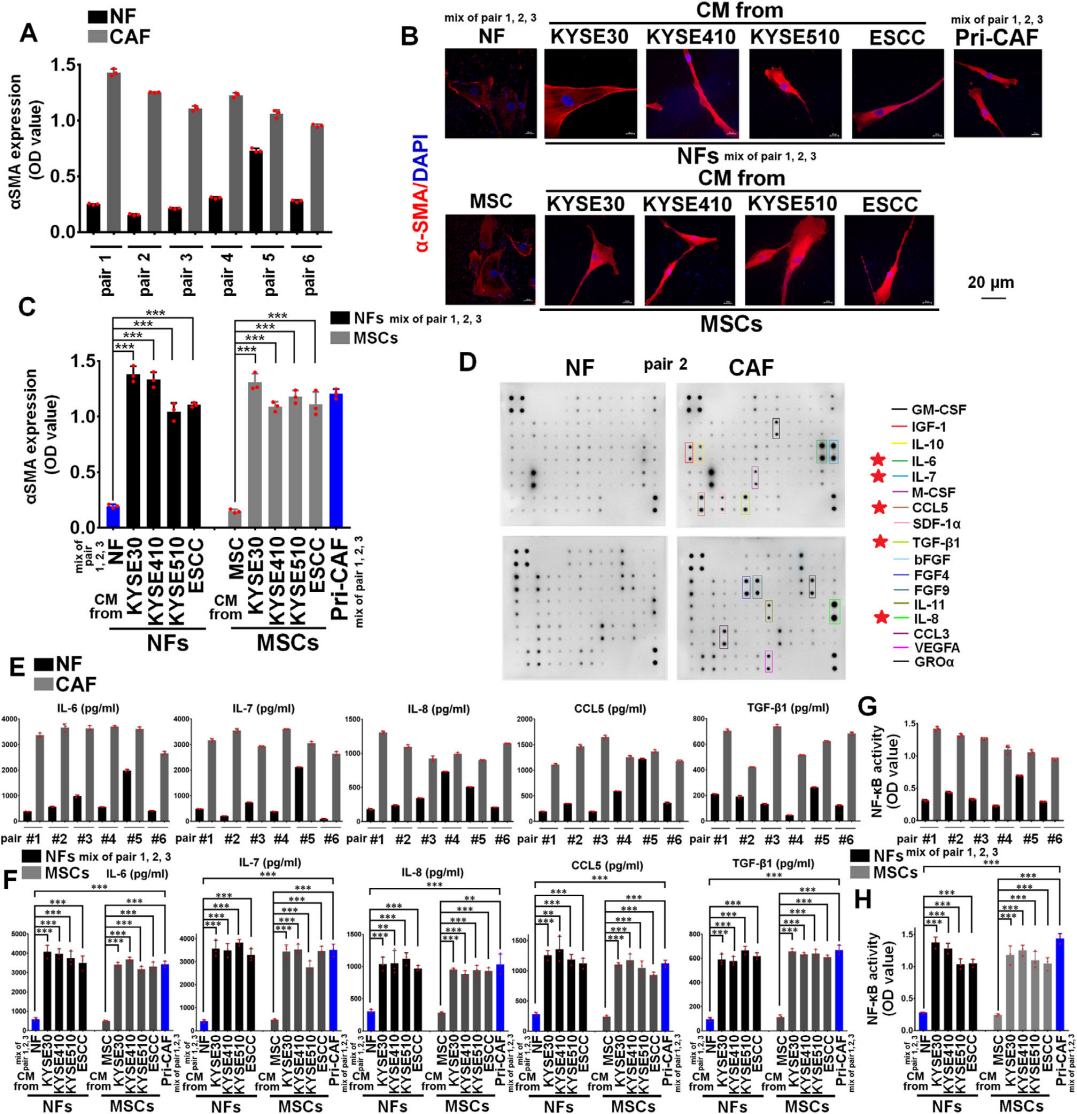
The expression profile of cytokines between NFs and CAFs. ELISA of αSMA levels in cell lysates of primary NFs and paired CAFs (six pairs). (B, C) NFs (a mixture of pairs 1, 2, and 3) or adipose‐derived MSCs cultured with the CM from KYSE30, KYSE410, KYSE510, or primary ESCC cells for 3 days. After the tumor CM was removed, NFs (a mixture of pairs 1, 2, and 3) and adipose‐derived MSCs were incubated with fresh RPMI1640 medium for 2 days, and fluorescent staining of αSMA in NFs, or adipose‐derived MSCs alone or these cells incubated with the CM from indicated ESCC cells. Scale bar, 20 μm as indicated (B). ELISA of αSMA levels in cell lysates of indicated stromal cells (C). Primary CAFs (a mixture of pairs 1, 2, and 3) were used as positive control. (D) The differential secreting status of cytokines in primary NF versus CAF from the same ESCC patient (pair 2), as assayed by cytokine antibody array. (E) ELISA assay showing the secretion of IL‐6, IL‐7, IL‐8, CCL5, and TGF‐β1 from six paired primary NFs and CAFs. (F) The experimental condition of (F) was consistent with that of (B). ELISA assay showing the secretion of IL‐6, IL‐7, IL‐8, CCL5, and TGF‐β1 from NFs (a mixture of pairs 1, 2, and 3) or adipose‐derived MSCs alone or incubated with the CM from indicated ESCC cells. Primary CAFs (a mixture of pairs 1, 2, and 3) were used as positive control. (G) Transcriptional factor activity assay of NF‐κB p65 activity in the nucleus of primary NFs and paired CAFs (six pairs). (H) The experimental condition of (H) was consistent with that of (B). NFs (a mixture of pairs 1, 2, and 3) or adipose‐derived MSCs cultured with the CM from indicated ESCC cells. NF‐κB p65 activity was examined using transcriptional factor activity assay. Primary CAFs (a mixture of pairs 1, 2, and 3) were used as positive control. **p < 0.01;***p < 0.001; two‐tailed unpaired Student's *t*‐test. Error bars represent mean ± SD of three independent experiments

The human ESCC cell lines were generously provided by Dr. Yutaka Shimada of Kyoto University, and characteristics of ESCC cell lines are listed in Tables S2 and S3. The culture condition of these cell lines was according to our previous reports.^17^, ^19^ One case of normal esophageal epithelial cell (NEEC) isolated from the adjacent nontumorous esophageal tissue (over 5 cm from the clinical stage II ESCC tissue^20^), one case of primary ESCC cell (clinical stage: II), and six paired primary CAFs and NFs isolated from six cases of clinical ESCC tissues (clinical stage: II) and their adjacent nontumorous esophageal tissues (over 5 cm from the tumor tissue^20^, ^21^), were obtained from CHI Scientific, Inc. Human adipose‐derived mesenchymal stem cells (MSCs; isolate from normal human adipose tissue) and lymphatic capillary endothelial cells (HLECs; isolate from normal human lymph node) were purchased from Sciencell, Inc. All primary cells were cultured with RPMI1640 medium containing 10% fetal bovine serum (FBS). Primary cells used in the present study are all listed in Table S3. Six paired primary NFs and CAFs were used to evaluate the expression of α smooth muscle actin (αSMA), the secretion of interleukin‐1β (IL‐6), IL‐7, IL‐8, CCL5, and transforming growth factor‐β1 (TGF‐β1), or the nuclear factor‐κB (NF‐κB) activity in Figure 1A, E, or G, respectively. Pair 2 primary NFs and CAFs was applied to antibody assay in Figure 1D. Primary NFs and paired CAFs (a mixture of pairs 1, 2, and 3) were, respectively, applied to the rest of *in vitro* assays. The mixture of pairs 1, 2, and 3 NFs‐activated CAFs (primed by KYSE30 cells) were used to xenografted models. The adipose‐derived MSCs were applied to confocal and immunoblotting assays (evaluation of αSMA expression), ELISA assays (assessment of IL‐6, IL‐7, IL‐8, CCL5, and TGF‐β1 secretions and the intracellular NF‐κB activity), as indicated. For establishing stable NOX5 small hairpin (sh) RNA ESCC cell lines and ESCC cell lines stably expressing NOX5 plasmid or Flag‐NOX5 Y476/478F mutant, the exact information and protocols were described previously.^17^ TNF‐α (Santa Cruz, Catalog number: sc‐37216‐SH) or IL‐1β (Santa Cruz, Catalog number: sc‐39615‐SH) shRNA was transiently transfected into KYSE30 and KYSE410 cells.

### 2.3 Preparation of conditioned media

**FIGURE 2:**
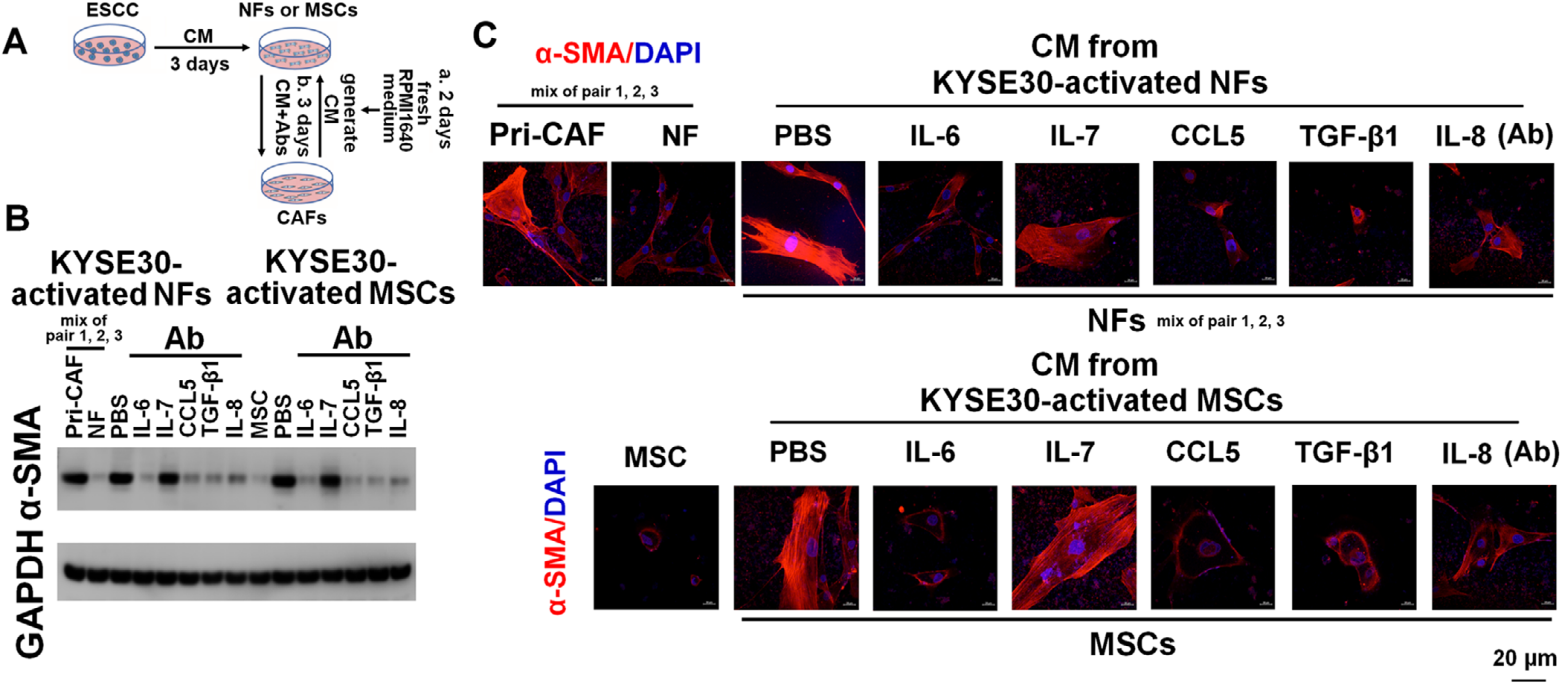
KYSE30 cells‐activated CAFs induce the activation of NFs and MSCs via secreting of various cytokines. NFs (a mixture of pairs 1, 2, and 3) and adipose‐derived MSCs were incubated with the CM from KYSE30 cells for 3 days. Then, the tumor CM was removed, the fresh RPMI1640 medium was added for 2 days to generate CM from activated CAFs. CM from NFs or adipose‐derived MSCs‐activated CAFs was further added to corresponding NFs (a mixture of pairs 1, 2, and 3) and adipose‐derived MSCs alone or in the presence of Abs of indicated cytokines for 3 days. The intracellular expression of αSMA was evaluated using immunoblotting (B) and confocal assay (C). Primary CAFs (a mixture of pairs 1, 2, and 3) were used as positive control. (B, C) NFs or adipose‐derived MSCs were cultured with the CM from corresponding NFs or adipose‐derived MSCs‐activated CAFs (primed by KYSE30 cells) alone or in the presence of several Abs, including IL‐6, IL‐7, IL‐8, CCL5, and TGF‐β1 (10 μg/ml). Immunoblotting (B), or fluorescent αSMA/DAPI staining (C) of αSMA in indicated stromal cells. Scale bar, 20 μm as indicated

**FIGURE 3:**
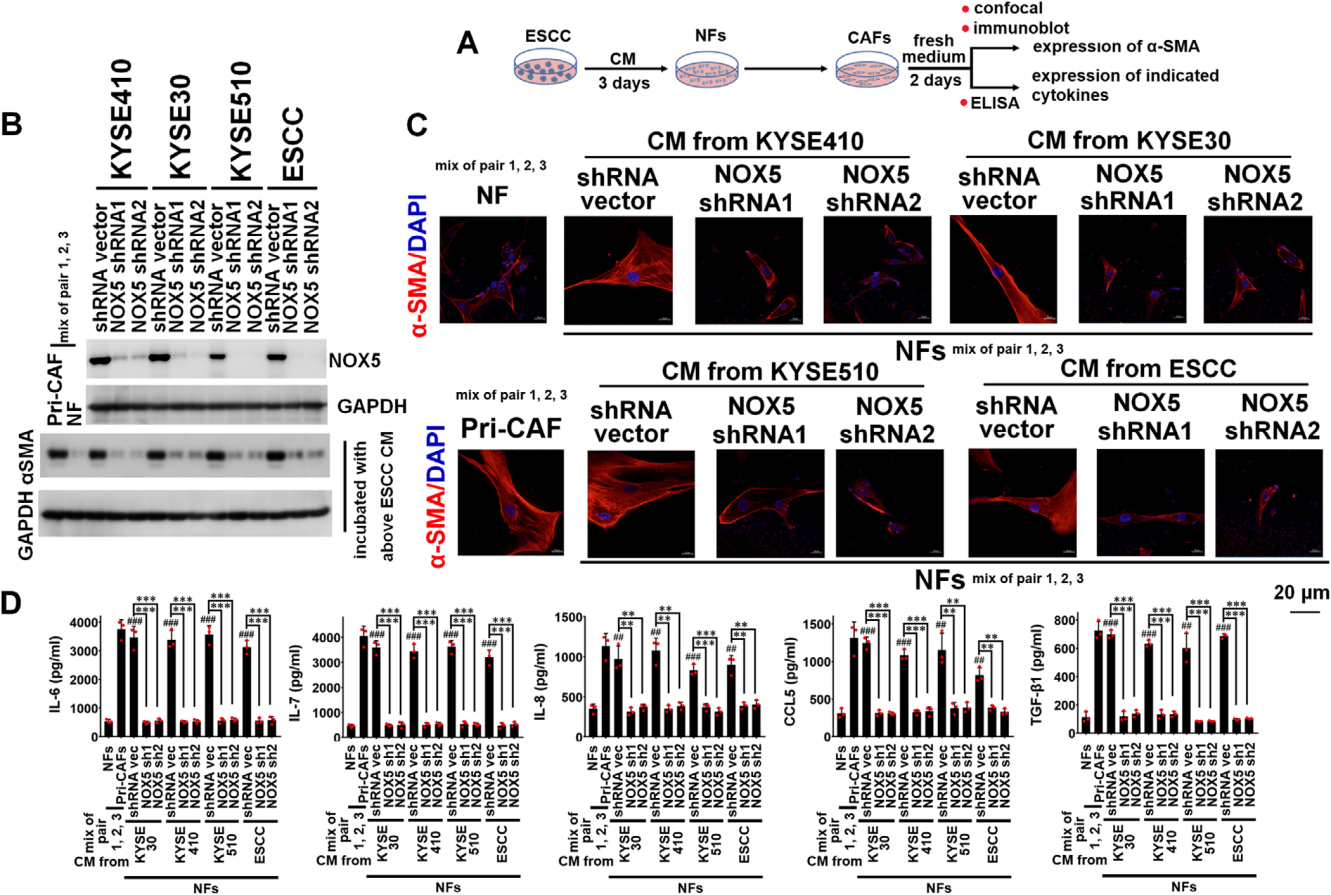
Intratumoral NOX5 is critically contributed to the activation of NFs into CAFs. NFs (a mixture of pairs 1, 2, and 3) were incubated with the CM from KYSE30, KYSE410, KYSE510, and primary ESCC cells harbored control or NOX5 shRNA for 3 days. Then, fibroblasts were cultured with fresh RPMI1640 medium for 2 days. The intracellular expression of αSMA was evaluated using immunoblotting (B) and confocal assay (C). The secretion of IL‐6, IL‐7, IL‐8, CCL5, and TGF‐β1 from fibroblasts was assessed using ELISA assay (D). Primary CAFs (a mixture of pairs 1, 2, and 3) were used as positive control. (B, C) Stably silencing NOX5 in the indicated ESCC cell lines and primary ESCC cells analyzed by immunoblotting. GAPDH was used as the internal control (B, upper panel). Immunoblotting (B, lower panel) or confocal (C) of αSMA levels in NFs or fibroblasts derived from NFs incubated with the CM from indicated ESCC cells harbored control shRNA or NOX5 shRNA. Scale bar, 20 μm as indicated. (D) ELISA assay showing the secretion of IL‐6, IL‐7, IL‐8, CCL5, and TGF‐β1 from NFs or fibroblasts derived from NFs incubated with the CM from indicated ESCC cells harbored control shRNA or NOX5 shRNA. # represents the statistical significance of NFs alone versus NFs treated with the CM from indicated ESCC cells harbored control shRNA. * represents the statistical significance of NFs treated with the CM from indicated ESCC cells harbored control shRNA versus NFs treated with the CM from indicated ESCC cells harbored NOX5 shRNA. ## *p* < 0.01; ### *p* < 0.001; ** *p* < 0.01; *** *p* < 0.001; two‐tailed unpaired Student's *t*‐test. Error bars, mean ± SD of three independent experiments

**FIGURE 4:**
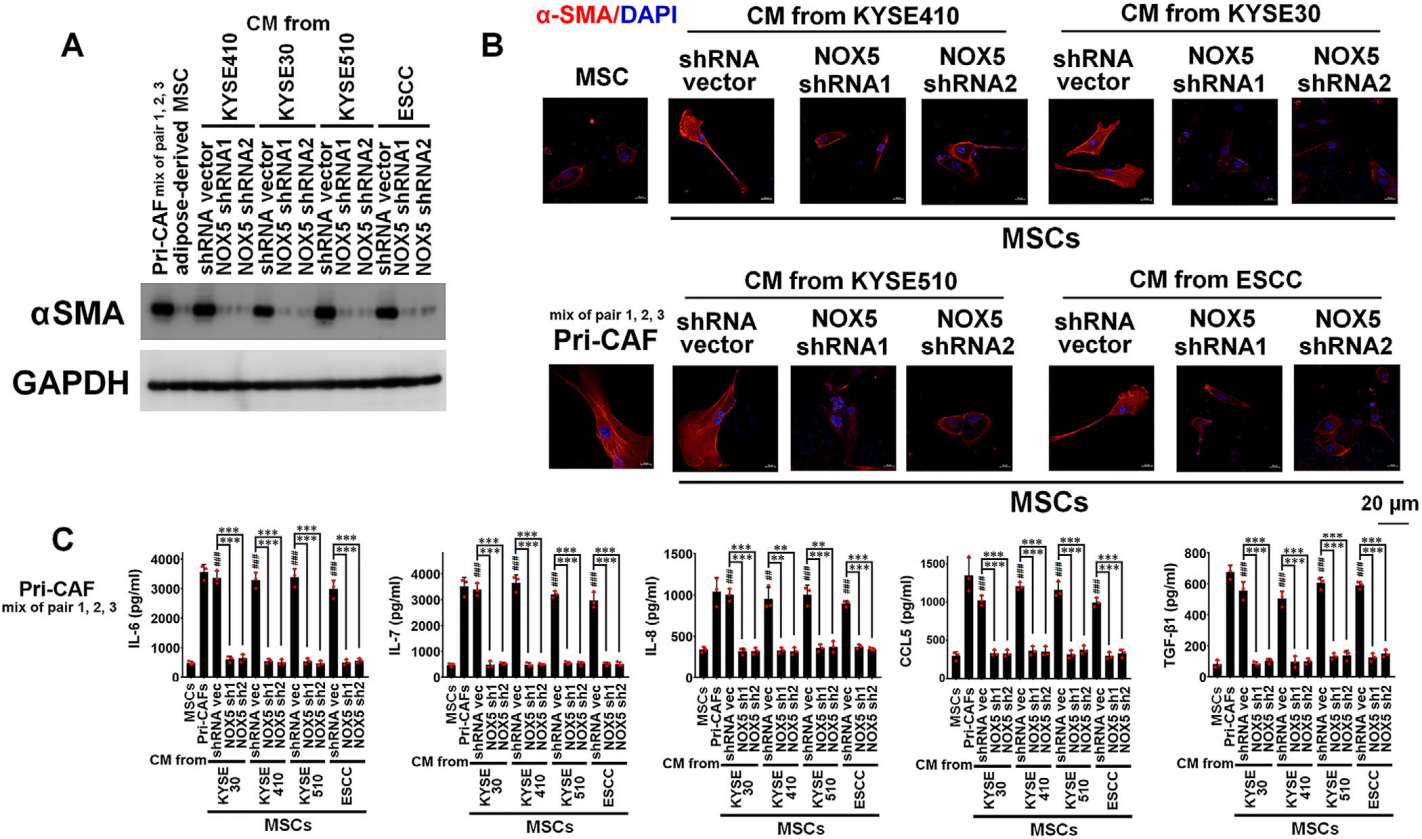
Intratumoral NOX5 is critically contributed to the activation of adipose‐derived MSCs into CAFs. The experimental condition of this figure was consistent with that of Figure 3. (A, B) Immunoblotting (A) or confocal (B) of αSMA levels in adipose‐derived MSCs, or fibroblasts derived from adipose‐derived MSCs incubated with the CM from KYSE410, KYSE30, KYSE510, or primary ESCC cells harbored control shRNA or NOX5 shRNA. Scale bar, 20 μm as indicated. (C) ELISA assay showing the secretion of IL‐6, IL‐7, IL‐8, CCL5, and TGF‐β1 from adipose‐derived MSCs and fibroblasts derived from adipose‐derived MSCs incubated with the CM from indicated ESCC cells harbored control shRNA or NOX5 shRNA. Primary CAFs (a mixture of pairs 1, 2, and 3) were used as positive control. # represents the statistical significance of adipose‐derived MSCs alone versus adipose‐derived MSCs incubated with the CM from indicated ESCC cells harbored control shRNA. *represents the statistical significance of adipose‐derived MSCs incubated with the CM from indicated ESCC cells harbored control shRNA versus adipose‐derived MSCs incubated with the CM from indicated ESCC cells harbored NOX5 shRNA. ### *p* < 0.001; **p < 0.01; ***p < 0.001; two‐tailed unpaired Student's *t*‐test. Error bars, mean ± SD of three independent experiments

**FIGURE 5:**
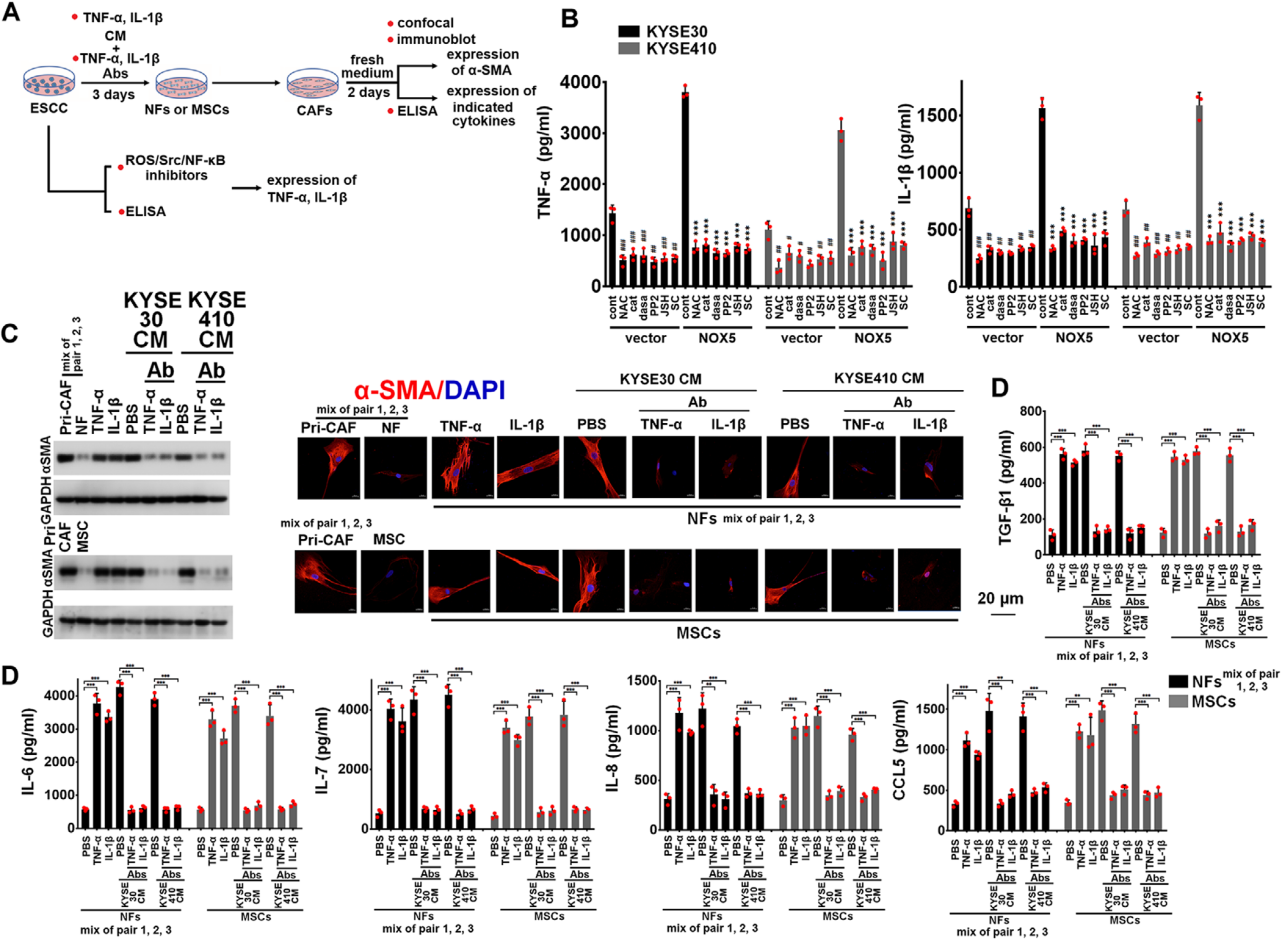
NOX5‐induced TNF‐α or IL‐1β mediates the activation of NFs and adipose‐derived MSCs into CAFs. Control or NOX5‐overexpressing KYSE30 and KYSE410 cells were treated with inhibitors of ROS/Src/NF‐κB pathway, and the secretion of TNF‐α and IL‐1β was evaluated using ELISA assay (B). NFs (a mixture of pairs 1, 2, and 3) or adipose‐derived MSCs were incubated with recombinant human TNF‐α or IL‐1β protein, or CM from KYSE30 and KYSE410 cells alone or in the presence of TNF‐α or IL‐1β Ab for 3 days. Then, fibroblasts were cultured with fresh RPMI1640 medium for 2 days. The intracellular expression of αSMA was evaluated using immunoblotting and confocal assay (C). The secretion of IL‐6, IL‐7, IL‐8, CCL5, and TGF‐β1 from fibroblasts was assessed using ELISA assay (D). Primary CAFs (a mixture of pairs 1, 2, and 3) were used as positive control. (B) Control vector or NOX5‐overexpressing KYSE30 (black bar) and KYSE410 (gray bar) cells were pretreated with ROS scavenger NAC (2 mM, pretreated with 90 min), or treated with H_2_O_2_ scavenger‐PEG‐catalase (400 units/ml), 100 nM dasatinib, 1 μM PP2, 5 μM JSH‐23, 5 μM SC75741, or control solvent. The concentration of TNF‐α and IL‐1β in CM from indicated ESCC cells was assayed by ELISA. # represents the statistical significance of indicated treatments versus control cells. * represents the statistical significance of indicated treatments versus NOX5‐overexpressing cells. # *p* < 0.05; ## *p* < 0.01; ### *p* < 0.001; ***p < 0.001; two‐tailed unpaired Student's *t*‐test. (C, D) NFs (a mixture of pairs 1, 2, and 3) or adipose‐derived MSCs were treated with recombinant TNF‐α or IL‐1β protein (10 ng/ml), or the CM from KYSE30 or KYSE410 cells in the presence or absence of the TNF‐α or IL‐1β Ab (10 μg/ml) for 3 days. Then, culture media were removed and added fresh RPMI1640 medium to these fibroblasts for 2 days. (C) Immunoblotting (left panel) or confocal assay (right panel) evaluating the expression of αSMA in NFs (a mixture of pairs 1, 2, and 3) or adipose‐derived MSCs. Scale bar, 20 μm as indicated. (D) ELISA assay was used to detect the secretion of IL‐6, IL‐7, IL‐8, CCL5, and TGF‐β1 from indicated stromal cells. Primary CAFs (a mixture of pairs 1, 2, and 3) were used as positive control. **p < 0.01; ***p < 0.001; two‐tailed unpaired Student's *t*‐test. Error bars, mean ± SD of three independent experiments

**FIGURE 6:**
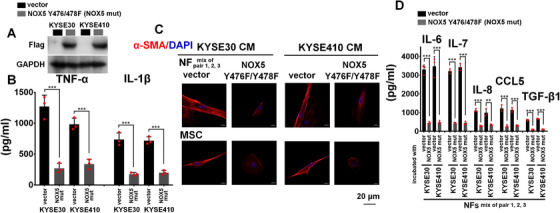
NOX5 Y476/478 sites are critical for NOX5‐mediated CAFs activation. The experimental condition of this figure was consistent with that of Figure 3. (A) Transfection of NOX5 Y476/478F plasmid into KYSE30 and KYSE410 cells. The transfection efficiency was evaluated using immunoblotting. GAPDH was used as the loading control. (B) The concentration of TNF‐α and IL‐1β in CM from KYSE30 and KYSE410 cells harbored control vector or NOX5 Y476/478F plasmid, was assayed by ELISA. (C) Confocal analysis of αSMA levels in NFs (a mixture of pairs 1, 2, and 3), adipose‐derived MSCs incubated with the CM from indicated ESCC cells harbored control vector or NOX5 Y476/478F plasmid. Scale bar, 20 μm as indicated. (D) ELISA assay showing the secretion of IL‐6, IL‐7, IL‐8, CCL5, and TGF‐β1 from NFs (a mixture of pairs 1, 2, and 3) and adipose‐derived MSCs incubated with the CM from KYSE30 and KYSE410 cells harbored control vector or NOX5 Y476/478F plasmid. **p < 0.01; ***p < 0.001; two‐tailed unpaired Student's *t*‐test. Error bars, mean ± SD of three independent experiments

KYSE30, KYSE410, KYSE510, and primary ESCC cells (Figures 2 and S2) or these cells harbored control or NOX5 shRNA (Figures 3 and 4), KYSE30, KYSE410 cells harbored control vector or NOX5 Y476/478F mutant cells (Figure 6), and KYSE30, KYSE410 cells harbored control, TNF‐α, or IL‐1β shRNA (Figure S7) were cultured with FBS‐free RPMI1640 medium for 2 days.^22^, ^23^ Then, the conditioned media (CM) from these indicated ESCC cells were collected to obtain tumor‐derived CM, which were used to observe the activation of NFs and adipose‐derived MSCs into CAFs. RPMI1640 medium containing 10% FBS was removed from NFs and adipose‐derived MSCs, and these cells were then cultured with above ESCC cells‐derived CM for 3 days. After the tumor CM was removed, fibroblasts were incubated with fresh FBS‐free RPMI1640 medium for 2 days, and the expression of αSMA in fibroblasts was evaluated using immunoblotting or confocal assays. The concentration of IL‐6, IL‐7, IL‐8, CCL5, and TGF‐β1 in culture medium of fibroblasts was assayed by ELISA. In addition to activate CAFs, the CM from above indicated KYSE30 and KYSE410 cells was used to analyze the secretion of TNF‐α and IL‐1β (Figures 5B and S7A). The CM from control or NOX5 overexpressing KYSE30 cells was applied to evaluate the cytokine profile (Figure S5B) and level of TNF‐α and IL‐1β (Figures 5B and S5C). In Figure 8, CM of activated CAFs (derive from a mixture of pairs 1, 2, and 3 NFs) primed by CM from KYSE30 and KYSE410 cells was added to corresponding KYSE30 or KYSE410 control or NOX5 shRNA cells, and the proliferation of tumor cells was measured by 3‐(4,5‐dimethylthiazol‐2‐yl)‐5‐(3‐carboxymethoxyphenyl)‐ 2‐(4‐sulfophenyl)‐2*H*‐tetrazolium, inner salt (MTS) assay. KYSE30 or KYSE410 cells‐activated CAFs were used to evaluate the invasive ability of KYSE30 or KYSE410 control or NOX5 shRNA cells, respectively. The function of indicated cytokines in conditioned medium was depleted by indicated neutralizing antibodies (Abs; the concentration of the indicated Ab was 10 μg/ml).

### 2.4 Cell proliferation assay (MTS assay)

KYSE30 and KYSE410 control or NOX5 shRNA cells were cultured in 96‐well plates (3×10^3^/well) in the absence or presence of CM from their corresponding parental KYSE30 and KYSE410 cells‐activated CAFs (derive from a mixture of pairs 1, 2, and 3 NFs; Figures 8B and S9A) or primary CAFs (a mixture of pairs 1, 2, and 3; Figure S9A) for 4 days. Growth ability of tumor cells was evaluated by the One Solution MTS Assay (Progema). This assay was repeated five to six times.

### 2.5 Cell invasion and migration assay

For invasion assay, KYSE30 and KYSE410 control or NOX5 shRNA cells (5 × 10^4^/well) were seeded into the upper chamber (Boyden chambers with 8 μm‐inserts) in 200 μl of FBS‐free RPMI1640 medium. The corresponding parental KYSE30 and KYSE410 cells‐activated CAFs (derive from a mixture of pairs 1, 2, and 3 NFs; Figures 8C and S9B) and primary CAFs (a mixture of pairs 1, 2, and 3; Figure S9B) (5 × 10^4^/well) were, respectively, placed in the lower chamber. Subsequent experimental procedures were according to our previous studies.^17^, ^19^

For migration assay, HLECs (1 × 10^4^/well) were seeded on the inserts of 96‐well Boyden chambers and incubated with KYSE30 and KYSE410 control or NOX5 shRNA cells‐activated fibroblasts (derive from a mixture of pairs 1, 2, and 3 NFs; Figures 8D and S9C), or primary CAFs (a mixture of pairs 1, 2, and 3; Figure S9C) (1 × 10^4^/well) in the lower compartment at 37°C for 16 h. Following experiments were according to instructions of CytoSelect 96‐well cell migration assay (Cell Biolabs; Cat# CBA‐106). These assays were repeated five times.

### 2.6 Confocal assay

NFs (a mixture of pairs 1, 2, and 3), adipose‐derived MSCs, activated CAFs, and primary CAFs (a mixture of pairs 1, 2, and 3) were fixed in 4% paraformaldehyde, and followed by incubating with primary αSMA antibody at 4°C for 12 h. Then, stromal cells were incubated with TRITC‐(tetramethyl rhodamine isothiocyanate)‐linked secondary antibody for 1 h at room temperature. DAPI (4′, 6‐diamidino‐2‐phenlindole; 0.1 μg/ml) was used to stain nuclei. Images were obtained using a confocal laser scanning microscope (Leica ST2, Leica, Germany).

### 2.7 Antibody array

For the secreted cytokine analysis, 500 μl of supernatants from the pair 2 primary NFs and CAFs (Figure 1D) and control or NOX5‐overexpressing KYSE30 cells (Figure S5B) were, respectively, subjected to antibody arrays against 120 unique cytokines (Raybiotech; Cat# AAH‐CYT‐1000). Each antibody of cytokines is spotted on membranes in duplicate vertically. Then, membranes were washed thrice with PBS, sequentially incubated with biotinylated antibodies, streptavidin‐horseradish peroxidase, and detection solutions. Membranes were exposed and analyzed the expression of cytokines.

### 2.8 ELISA assay

TNF‐α (Catalog number: ELH‐TNFα1) and IL‐1β (Catalog number: ELH‐ILβ1) ELISA kits were purchased from Raybiotech, and IL‐6 (Catalog number: D6050), IL‐7 (Catalog number: HS750), IL‐8 (Catalog number: D8000C), CCL5 (Catalog number: DRN00B), and TGF‐β1 (Catalog number: DB100B) ELISA kits were all obtained from R&D systems and used to evaluate the secretion of these cytokines.

**FIGURE 7:**
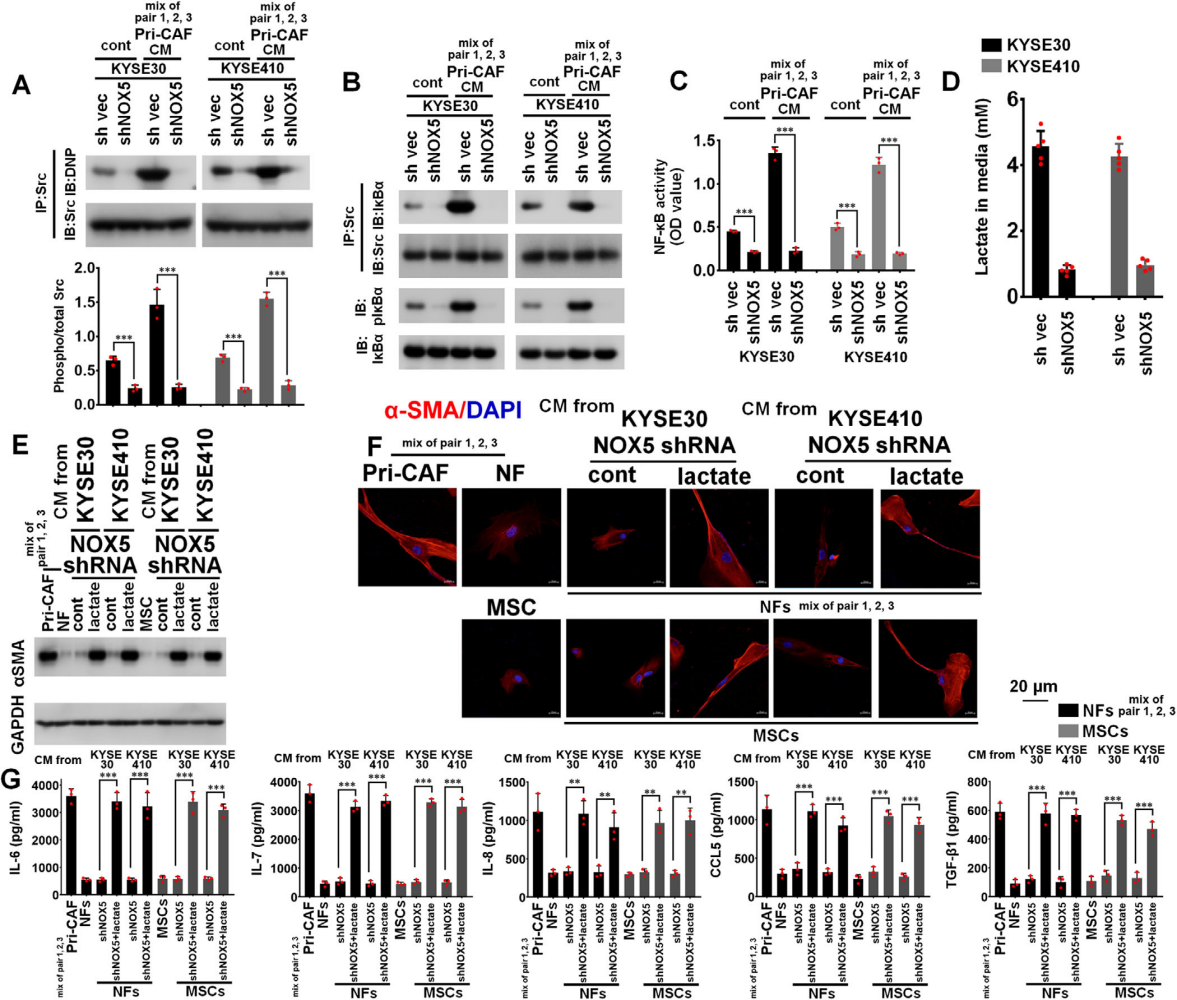
NOX5 mediates NF‐κB activation via controlling the interaction between Src and IκBα, and prompts lactate secretion from tumor cells. (A) KYSE30 and KYSE410 control or NOX5 shRNA cells were cultured with or without the CM from primary CAFs (a mixture of pairs 1, 2, and 3). Oxidized Src levels were measured using a modified OxyBlot protein detection kit (upper panel). The Src activity was assayed by Src activation quantitative ELISA assay (lower panel). (B) The experimental condition of (B) was consistent with that of (A). The lysates of indicated KYSE30 and KYSE410 cells were immunoprecipitated with Src antibody, and then immunoblotted using Src and IκBα antibodies (upper panel). The lysates of indicated cells were immunoblotted using IκBα and pIκBα (Tyr^42^) antibodies (lower panel). (C) The experimental condition of (C) was consistent with that of (A). The NF‐κB p65 activity in indicated KYSE30 and KYSE410 cells was assessed using transcriptional factor activity assay. (D) Extracellular lactate level was evaluated using lactate detection assay kit. (E–G) NFs (a mixture of pairs 1, 2, and 3) and adipose‐derived MSCs were incubated with the CM from KYSE30 and KYSE410 NOX5 shRNA cells alone or in the presence of 5 mM lactate for 3 days. After tumor CM and lactate were removed, stromal cells were cultured with fresh RPMI1640 medium for 2 days. Primary CAFs (a mixture of pairs 1, 2, and 3) were used as positive control. Immunoblotting (E) or confocal (F) of αSMA levels in stromal cells. Scale bar, 20 μm as indicated. (G) ELISA assay showing the secretion of IL‐6, IL‐7, IL‐8, CCL5, and TGF‐β1 from stromal cells. **p < 0.01; ***p < 0.001; two‐tailed unpaired Student's *t*‐test. Error bars, mean ± SD of three to five independent experiments

For evaluation of the activation status of Src (pSrc Tyr^419^/total Src ratio), control or NOX5‐depleted KYSE30 or KYSE410 cells alone or in the presence of CM from primary CAFs (a mixture of pairs 1, 2, and 3) were collected to obtain cell lysates. The expression of intratumoral pSrc Tyr^419^ and Src was analyzed using the human phospho‐Src (Tyr^419^) ELISA kit (Raybiotech; Catalog number: PEL‐SRC‐Y419‐T). Src‐IκBα ELISA binding assay was applied to quantify the interaction between Src and IκBα, as previously described with modifications.^24^ The cell culture condition of Src‐IκBα ELISA binding assay was consistent with that of Src activity assay. Lysates from indicated KYSE30 or KYSE410 cells were immunoprecipitated by Src antibody (IP: Src) and sepharose beads. Then, bound Src or IκBα released from beads was quantified using human Src or IκBα ELISA kit (Raybiotech; Catalog number: ELH‐SRC‐1 or ELH‐IKBA‐1), respectively. The intracellular protein expression of αSMA in six paired primary NFs and CAFs (Figure 1A), or NFs (a mixture of pairs 1, 2, and 3), adipose‐derived MSCs, these two types of cells incubated with the CM from KYSE30, KYSE410, KYSE510, and primary ESCC cells, or primary CAFs (a mixture of pairs 1, 2, and 3) (Figure 1C) was evaluated by the human αSMA ELISA kit (Cloud‐Clone; Catalog number: SEB342Mi). The NF‐κB p65 transcriptional activity in six paired NFs and CAFs (Figure 1G), NFs (a mixture of pairs 1, 2, and 3), adipose‐derived MSCs, activated CAFs (primed by CM from KYSE30, KYSE410, KYSE510, and primary ESCC cells), and primary CAFs (a mixture of pairs 1, 2, and 3) (Figure 1H), and indicated KYSE30 or KYSE410 cells incubated with CM from primary CAFs (a mixture of pairs 1, 2, and 3) (Figure 7C) was assessed by human NF‐κB transcriptional activity ELISA kit (Raybiotech; Cat# TFEH‐p65). These assays were repeated three to five times.

### 2.9 Immunoblotting

Lysates from indicated stromal cells and KYSE30, KYSE410, KYSE510, or primary ESCC cells were resolved using SDS‐PAGE, transferred to PVDF membranes, and, respectively, incubated with primary antibodies against αSMA (evaluation of the activation of NFs and adipose‐derived MSCs to CAFs), NOX5 (evaluation of the NOX5 expression in KYSE30, KYSE410, KYSE510, or primary ESCC cells harbored control or NOX5 shRNA in Figure 3B, assessment of NOX5 level in KYSE30 or KYSE410 transfected with control vector or NOX5 plasmid in Figure S5A), or Src, IκBα, pIκBα (assessment of the expression of these proteins in KYSE30, or KYSE410 cells harbored control or NOX5 shRNA alone or in the presence of CM from a mixture of pairs 1, 2, and 3 primary CAFs in Figures 7B and S8A) or Flag (Figure 6A). Subsequent experimental procedures were according to our previous study.^19^

### 2.10 RT‐PCR assay

RNA was isolated from NEEC, primary ESCC cells, and indicated ESCC cell lines. The protocols of RNA extraction, RT‐PCR, and the sequence of *GAPDH* were referred to our previous study.^19^ The primer sequences of *NOX5* were as follows: *NOX5*: 5′‐ACTCAGCAGTTTAAGACCATTGC‐3′ and 5′‐GGACTCTTTCACATGCAGAGC‐3′. This assay was repeated three times.

### 2.11 Src protein oxidation assay

OxyBlot protein detection kit (Abcam; Catalog number: ab178020) was applied to evaluate the oxidation of Src protein in control or NOX5‐depleted KYSE30 or KYSE410 cells alone or in the presence of CM from primary CAFs (a mixture of pairs 1, 2, and 3). Briefly, cell lysates were obtained using 1 × extraction buffer, and then immunoprecipitated with primary Src antibody and protein A/G sepharose beads. The released immune complex was incubated with 2, 4‐dinitrophenylhydrazine (DNPH) reagent at room temperature for 15 min, and subjected to immunoblotting. The oxidized Src on PVDF membranes was detected using 1 × primary anti‐DNP antibody.

### 2.12 Measurement of lactate production

Colorimetric lactate kit (Nanjing Jiancheng Bioengineering Institute) was applied to measure the lactate secretion from control or NOX5 shRNA transfected KYSE30 and KYSE410 cells. Cells were seeded in 24‐well plates (2×10^5^/well), and aliquots of media from each well were assessed 24 h later for the amount of lactate. This assay was repeated five times.

### 2.13 Immunohistochemistry analysis of clinical ESCC tissue samples

Collections of paraffin‐embedded ESCC samples were authorized by the Institutional Review Board of Peking University Cancer Hospital. For immunohistochemistry (IHC) assay, after antigen retrieval in Tris‐EDTA buffer (pH 9.0) was carried out, ESCC samples were blocked by PBS with 5% BSA, and then incubated with IL‐6, IL‐7, IL‐8, CCL5, TGF‐β1, NF‐κB p65, NOX5, pIκBα, pSrc, TNF‐α, and IL‐1β antibodies (antibodies were diluted at 1:100, except NF‐κB p65 diluted at 1:500, pSrc antibody diluted at 1:200) overnight at 4°C. Subsequently, secondary antibody was added, and immunodetection was performed using DAB (Dako). The staining index (SI) calculation was described previously.^17^

### 2.14 Xenograft studies

KYSE30 shRNA vector or NOX5 shRNA cells (2×10^6^/mouse) and activated CAFs (6×10^6^/mouse) were coinjected into the flank of mouse to observe the effect of CAFs on the progression of ESCC tumors. The mouse strain used in this experiment is nonobese diabetic/severe combined immunodeficiency (NOD/SCID) mouse (*n* = 5/group; female; 4 weeks old). For antibody treatment, mice were intravenously injected with IL‐6, IL‐7, IL‐8, or TGF‐β1 Ab (10 μg/mouse twice times per week, i.v.) after the xenografts reached at approximately 80–100 mm^3^. Animals were euthanized 33 days after ESCC cell implantation. Then, tumors were dissected and the formula: (Length × Width^2^) × 0.5 was applied to assess the tumor volumes. CAFs‐mediated lymphatic metastases of KYSE30 cells were evaluated using the popliteal lymph node metastasis model. The indicated KYSE30 cells and activated CAFs were coinjected into the foot pad of animals. Other experimental conditions in present model were similar with those of above animal model. Groups of animal models were categorized as follows: KYSE30 cells shRNA vector alone, KYSE30 cells shRNA vector + CAFs alone or in the presence of IL‐6, IL‐7, IL‐8, or TGF‐β1 Ab, KYSE30 cells NOX5 shRNA alone, KYSE30 cells NOX5 shRNA vector + CAFs.

### 2.15 Statistical analysis

Graphpad Prism 7.0 (GraphPad Software Inc, La Jolla, CA, USA) was applied to conduct all analyses in the present study. The statistical analysis between two groups used Student's *t*‐test (two‐tailed). Correlation analysis between two factors of clinical tissue samples was carried out using chi‐square test. Data are presented as the mean ± standard deviation (SD). *p*‐value of < 0.05 meant that the results were statistically significant.

## 3 RESULTS

### 3.1 Comprehensive cytokine analyses of CAFs activated by NOX5‐positive ESCC show a unique secreting pattern

Six paired NFs and CAFs were applied to evaluate the expression of αSMA, which is often used as a marker for CAFs. As shown in Figure 1A, the protein level of αSMA was evidently higher in CAFs than in paired NFs. Our previous study has demonstrated that NOX5 protein was highly expressed in ESCC tissues and cell lines.^17^ Results of Figure S1 showed that the mRNA level of *NOX5* was markedly increased in eight ESCC cell lines and one primary ESCC cells, compared with one primary NEEC. Then, we evaluated whether NOX5‐positive ESCC cells, such as KYSE30, KYSE410, KYSE510, or primary ESCC cells, could induce the activation of NFs to CAFs.^17^ NFs (a mixture of pairs 1, 2, and 3) were incubated with CM from KYSE30, KYSE410, KYSE510, or primary ESCC cells. Results of confocal (Figure 1B) and ELISA assay (Figure 1C) revealed that αSMA expression was markedly increased in NFs‐incubated with the CM of ESCC cells.

We next analyzed the cytokine profiles of paired NFs and CAFs (pair 2) using Raybio Human Cytokine antibody array, and found that IL‐6, IL‐7, IL‐8, CCL5, and TGF‐β1 were significantly higher in primary CAF, compared with paired NF (Figure 1D). ELISA assay for evaluating the release of IL‐6, IL‐7, IL‐8, CCL5, and TGF‐β1 from six paired NFs and CAFs confirmed the results of antibody array (Figure 1E). As Figure 1F shown, secretions of IL‐6, IL‐7, IL‐8, CCL5, and TGF‐β1 were increased in NOX5‐positive ESCC cells‐activated CAFs. The secretion of these identified cytokines from fibroblasts was positively correlated with the expression of αSMA (Figure 1A, B, C, E, and F).

A recent study has shown that adipose tissue can promote the malignant progression of ESCC.^25^ Adipose tissues‐derived MSCs may possibly been activated to CAFs in several types of tumors. We examined whether NOX5‐positive ESCC cells could activate adipose tissues‐derived MSCs to CAFs. Results of Figure 1B, C, and F showed that the level of αSMA was evidently upregulated in adipose‐derived MSCs following incubation with the CM from KYSE30, KYSE410, KYSE510, or primary ESCC cells (Figure 1B and C), and IL‐6, IL‐7, IL‐8, CCL5, and TGF‐β1 were strongly secreted from adipose‐derived MSCs incubated with the CM from these indicated ESCC cells (Figure 1F). Thus, NOX5‐positive ESCC cells induce NFs but also adipose‐derived MSCs to acquire CAF features and stimulate the secretion of cytokines from these cells.

We further evaluated whether CAFs activated by NOX5‐positive ESCC cells could reprogram NFs and adipose‐derived MSCs into CAFs (Figure 2A). The expression of αSMA in NFs (a mixture of pairs 1, 2, and 3) and adipose‐derived MSCs was induced after stimulation with the CM from their respective parental cell‐derived CAFs, which were activated by KYSE30 cells (Figure 2B and C). Furthermore, the αSMA expression in NFs and adipose‐derived MSCs was suppressed when the CM from activated CAFs was cotreated with IL‐6, IL‐8, CCL5, or TGF‐β1 Ab (10 μg/ml), but not when the CM was cotreated with IL‐7 Ab (10 μg/ml) (Figure 2B and C). Similar results were also obtained in KYSE410, KYSE510, or primary ESCC cells‐activated CAFs (Figure S2A–S2C).

### 3.2 NF‐κB is activated in CAFs and correlated with the levels of cytokines

NF‐κB is known to stimulate the expression of various cytokines.^26^, ^27^ We analyzed the activity of NF‐κB and its correlation with the identified cytokines in the six paired NFs and CAFs. Results of Figure 1A, E, and G showed that the transcriptional activity of NF‐κB was higher in primary CAFs than that of paired NFs (six pairs), and NF‐κB activity was tightly correlated with the expression of αSMA and above identified cytokines. Furthermore, NF‐κB activity was higher in ESCC cells‐activated CAFs than its parental NFs (a mixture of pairs 1, 2, and 3) or adipose tissue‐derived MSCs (Figure 1H), and correlated with the expression of αSMA and secretions of indicated cytokines (Figure 1B, C, F, and H). JSH‐23 or SC75741 (inhibition of NF‐κB transcriptional activity; 5 μM) markedly suppressed the secretion of IL6, IL‐7, IL‐8, CCL5, and TGF‐β1 from the CAFs activated by the CM from KYSE410 or KYSE30 cells (Figure S3A–S3E). IHC staining and correlation analysis further showed that stromal NF‐κB was positively associated with the level of stromal IL‐6, IL‐7, IL‐8, CCL5, and TGF‐β1 in clinical ESCC samples (Figure S4).

### 3.3 NOX5 activates CAFs via stimulating TNF‐α or IL‐1β secretion from ESCC cells

We next investigated whether NOX5 expression in ESCC cells is critical for the activation of CAFs (Figure 3A). We found that the αSMA expression was strongly upregulated in NFs (a mixture of pairs 1, 2, and 3) or adipose‐derived MSCs after incubation with the CM from the control KYSE410, KYSE30, KYSE510, or primary ESCC cells, but not from NOX5‐depleted ESCC cells (Figures 3B, 3C, 4A, and 4B). ELISA results showed that the secretion of the indicated cytokines from CAFs activated by the CM from control ESCC cells were significantly higher than those from NFs or adipose‐derived MSCs incubated with the CM from NOX5‐depleted ESCC cells (Figures 3D and 4C). The secretion of cytokines from NFs or adipose‐derived MSCs incubated with NOX5‐depleted ESCC cells was almost similar with that of NFs or adipose‐derived MSCs alone (Figures 3D and 4C).

Because tumor‐derived cytokines are crucial for the activation of CAFs,^3^, ^18^ we screened which cytokines secreted from ESCC cells were under the control of NOX5 using cytokine antibody array. As shown in Figure S5A and S5B, secretion of several cytokines was significantly increased from NOX5‐overexpressing KYSE30 cells. Among them, TNF‐α and IL‐1β were most significantly upregulated. Results of ELISA assay confirmed those of cytokine antibody array, and further showed that overexpression or knockdown NOX5 markedly upregulated or downregulated the secretion of TNF‐α and IL‐1β from KYSE30 and KYSE410 cells (Figure S5C). We evaluated whether the secretion of TNF‐α or IL‐1β was dependent on NOX5‐controlled ROS signaling pathway (Figure 5A). Results of Figure 5B showed that inhibition of ROS/Src/NF‐κB pathway by N‐Acetyl‐L‐cysteine (NAC, ROS scavenger, 2 mM, pretreated with 90 min), PEG‐catalase (hydrogen peroxide (H_2_O_2_) scavenger, 400 units/ml), 100 nM dasatinib, 1 μM PP2 (Src inhibitors), 5 μM JSH‐23, or 5 μM SC75741, markedly decreased NOX5‐stimulated TNF‐α or IL‐1β secretion. As shown in Figure S6, the level of NOX5 in clinical ESCC samples was significantly correlated with the expression of pSrc, pIκBα, NF‐κB, TNF‐α, or IL‐1β.

We investigated whether TNF‐α or IL‐1β recapitulated the stimulatory effect of NOX5 on stromal cells. As shown in Figure 5C, the expression of αSMA was effectively induced in NFs (a mixture of pairs 1, 2, and 3) and adipose‐derived MSCs following stimulation with recombinant human TNF‐α or IL‐1β protein (10 ng/ml) or CM from KYSE30 or KYSE410 cells. Conversely, the blockage of the activity of TNF‐α, or IL‐1β in the CM of NOX5‐positive ESCC cells by TNF‐α, or IL‐1β Ab (10 μg/ml) substantially inhibited the expression of αSMA in stromal cells (Figure 5C). Correspondingly, NFs or adipose‐derived MSCs incubated with recombinant human TNF‐α or IL‐1β protein (10 ng/ml) or the CM from indicated ESCC cells could secrete higher levels of IL6, IL‐7, IL‐8, CCL5, and TGF‐β1, compared with NFs or MSCs alone (Figure 5D). TNF‐α or IL‐1β Ab (10 μg/ml) effectively blocked the secretion of the indicated cytokines from CAFs activated by the CM from KYSE410 and KYSE30 cells (Figure 5D). Inhibition of the secretion of TNF‐α and IL‐1β from KYSE30 and KYSE410 cells by shRNA effectively blocked these ESCC cells‐induced activation of CAFs (Figure S7A–S7C).

The Y476/478 sites in the NOX5 catalytic domain are critical for the activation of the ROS/Src pathway.^17^ Thus, we evaluated whether NOX5 Y476/478 mediated the secretion of TNF‐α or IL‐1β from ESCC cells to induce the activation of CAFs. After function‐loss NOX5 Y476/478F mutant was transfected into KYSE30 and KYSE410 cells, the secretion of TNF‐α or IL‐1β from indicated ESCC cells was evidently downregulated (Figure 6A and B). Correspondingly, the CM from KYSE30 and KYSE410 cells that harbored NOX5 Y476/478F plasmid could not induce the activation of NFs and adipose‐derived MSCs to CAFs (Figure 6C) and stimulate the secretion of IL6, IL‐7, IL‐8, CCL5, and TGF‐β1 from fibroblasts (Figure 6D). These results suggest that intratumoral NOX5‐activated ROS/Src/NF‐κB/cytokines axis is responsible for the transition of stromal cells to CAFs.

### 3.4 NOX5 activates intratumoral NF‐κB signaling via oxidizing and activating Src

Since Src was shown to be oxidized and activated by NOX5‐derived ROS in ESCC cells,^17^ we evaluated whether NOX5 can stimulate Src activity in the context of CAFs. As shown in Figure 7A, the CM from primary CAFs (a mixture of pairs 1, 2, and 3) stimulated Src oxidization and activation in KYSE30 or KYSE410 control shRNA cells. Conversely, NOX5 shRNA blocked the CAFs‐mediated oxidization and activation of Src (Figure 7A).

The phosphorylation of IκBα at Tyr^42^ site by Src contributes to the activation of NF‐κB. We found that inhibition of NOX5 expression effectively disrupted the interaction between Src/IκBα, suppressed the phosphorylation of IκBα Tyr^42^, and blocked the activity of NF‐κB in KYSE30 or KYSE410 cells incubated with the CM from primary CAFs (a mixture of pairs 1, 2, and 3) (Figures 7B, 7C, S8A, and S8B). These results suggest that NOX5 is essential for the association of IκBα with Src, which is required for NF‐κB activation.

### 3.5 NOX5‐derived lactate contributes to the activation of CAFs

Because lactate contributes to stroma reprogram, and ROS increases the lactate secretion from tumor cells,^28^, ^29^ we examined whether NOX5 could affect the concentration of extracellular lactate. As shown in Figure 7D, depletion of NOX5 in KYSE30 and KYSE410 cells significantly suppressed the level of lactate in ESCC CM. Furthermore, adding 5 mM lactate to the CM from NOX5‐depleted KYSE30 and KYSE410 cells could effectively induce the activation of NFs (a mixture of pairs 1, 2, and 3) or adipose‐derived MSCs to CAFs (Figure 7E and F) and enhance the secretion of IL‐6, IL‐7, IL‐8, CCL5, and TGF‐β1 from stromal cells, as compared with stromal cells incubated with the CM from NOX5‐depleted ESCC cells alone (Figure 7G).

### 3.6 NOX5 expression in ESCC cells facilitates CAFs‐mediated tumor malignancy *in vitro*

**FIGURE 8:**
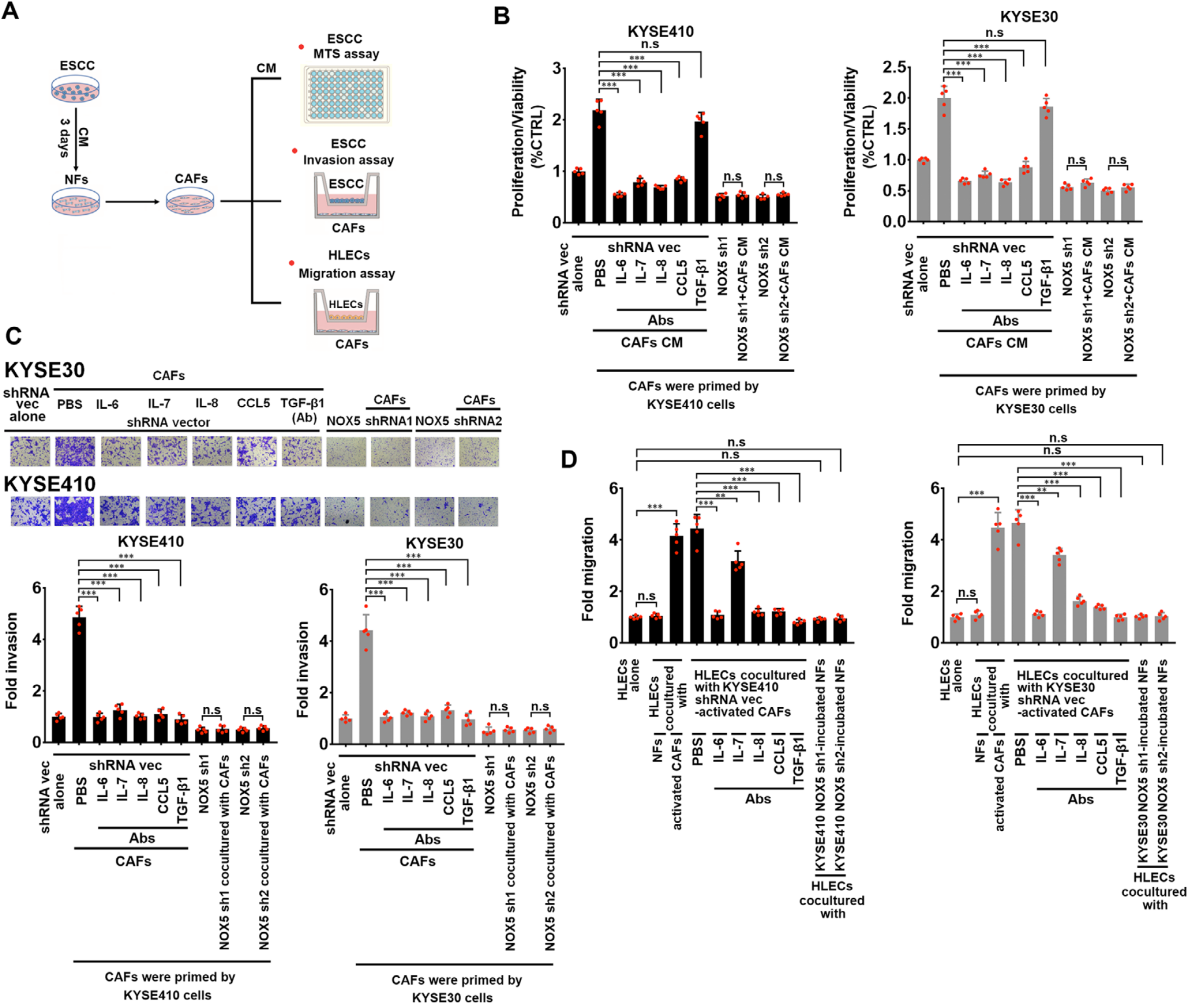
Activated CAFs assist ESCC malignant progression *in vitro*. NFs (a mixture of pairs 1, 2, and 3) were incubated with the CM from KYSE30 or KYSE410 cells for 3 days. Then, the tumor CM was removed, the fresh RPMI1640 medium was added to fibroblasts for 2 days to generate CM for MTS assay, and the activated CAFs were applied to Transwell invasion assay. KYSE30 or KYSE410 harbored control shRNA or NOX5 shRNA cells were incubated with the CM from corresponding parental cells‐activated CAFs. The growth ability was evaluated using MTS assay (B). CAFs regulated the invasion of ESCC cells was assessed by Transwell invasion assay. The activated CAFs primed by KYSE30 or KYSE410 cells were plated in the lower chamber. KYSE30 or KYSE410 harbored control shRNA or NOX5 shRNA cells (corresponding to their respective parental cells‐activated CAFs) were placed in the upper chamber (C). For evaluation of CAFs‐induced HLECs migration, NFs (a mixture of pairs 1, 2, and 3) were incubated with the CM from KYSE30 or KYSE410 harbored control shRNA or NOX5 shRNA cells for 3 days. Then, the tumor CM was removed, the fresh RPMI1640 medium was added to fibroblasts for 2 days, and these fibroblasts were used for subsequent assay. Migration of HLECs was evaluated using Transwell migration assay. Fibroblasts primed by KYSE30 or KYSE410 harbored control shRNA or NOX5 shRNA cells were plated in the lower chamber. HLECs were seeded in the upper chamber (D). (B) Growth rates of KYSE30 or KYSE410 cells harbored control shRNA incubated with the CM from corresponding parental cells‐activated CAFs alone or in the presence of IL‐6, IL‐7, IL‐8, CCL5, or TGF‐β1 Ab (10 μg/ml), or KYSE30 or KYSE410 cells harbored NOX5 shRNA alone or incubated with CM from their corresponding parental ESCC cells for 4 days. Cell growth was assayed by MTS assay. (C) Boyden chamber assay for KYSE30 or KYSE410 cells harbored control shRNA plated on the upper cell culture inserts with their corresponding parental ESCC cells‐activated CAFs in lower chambers alone or in the presence of IL‐6, IL‐7, IL‐8, CCL5, or TGF‐β1 Ab (10 μg/ml), or KYSE30 or KYSE410 cells harbored NOX5 shRNA plated on the upper cell culture inserts with or without their corresponding parental ESCC cells‐primed CAFs in lower chambers. (D) CytoSelect 96‐well cell migration assay for HLECs plated on upper cell culture inserts with NFs (a mixture of pairs 1, 2, and 3), NFs (a mixture of pairs 1, 2, and 3)‐activated CAFs (primed by KYSE30 or KYSE410 cells harbored control shRNA) alone or in the presence of IL‐6, IL‐7, IL‐8, CCL5, or TGF‐β1 Ab (10 μg/ml), or NFs (a mixture of pairs 1, 2, and 3) primed by the CM from KYSE30 or KYSE410 cells harbored NOX5 shRNA in lower chambers. n.s. no significant difference; *p < 0.05; ***p < 0.001; two‐tailed unpaired Student's *t*‐test. Error bars, mean ± SD of five independent experiments

We assessed whether ESCC‐activated CAFs reciprocally induced the progression of ESCC cells (Figure 8A). The results presented in Figure 8B showed that the proliferative rates of the KYSE30 and KYSE410 cells stimulated by the CM from activated CAFs were higher than those of KYSE30 and KYSE410 cells alone, as evaluated using MTS assay. The malignant proliferation of the KYSE30 and KYSE410 cells stimulated by the activated CAFs was significantly inhibited when the CM from activated CAFs was cotreated with IL‐6, IL‐7, IL‐8, or CCL5 Ab (10 μg/ml) (Figure 8B). We further determined whether the intratumoral NOX5 was contributed to CAFs‐induced growth of ESCC cells. The results presented in Figure 8B showed that activated CAFs could not promote the growth of NOX5‐knockdown KYSE30 and KYSE410 cells, as compared with that of NOX5‐depleted ESCC cells alone.

We used Boyden chambers assay to examine the invasiveness of KYSE30 and KYSE410 cells by plating them on matrigel‐coated inserts with their corresponding parental cells‐activated CAFs in the lower wells. Activated CAFs could significantly induce the invasion of ESCC cells, whereas the invasion of KYSE30 and KYSE410 cells was largely inhibited when IL‐6, IL‐7, IL‐8, CCL5, or TGF‐β1 Ab (10 μg/ml) was added to the coculture transwell system (Figure 8C). However, activated CAFs could not induce the invasion of indicated ESCC cells that harbored NOX5 shRNA (Figure 8C).

Because the lymphatic metastasis is the crucial step in ESCC progression,^30^, ^31^ we further evaluated the effect of CAFs on metastasis of HLECs *in vitro*. Results of Figure 8D showed that HLECs migration was markedly stimulated by fibroblasts primed by control KYSE30 and KYSE410 cells but not by NOX5‐knockdown ESCC cells. IL‐6, CCL5, TGF‐β1, IL‐7, and IL‐8 Abs (10 μg/ml) effectively inhibited CAFs‐enhanced migration of HLECs (Figure 8D). Primary CAFs (a mixture of pairs 1, 2, and 3) could produce similar effects on tumor malignancy as activated CAFs (Figure S9A–S9C).

### 3.7 CAFs assist tumor malignant progression *in vivo*

**FIGURE 9:**
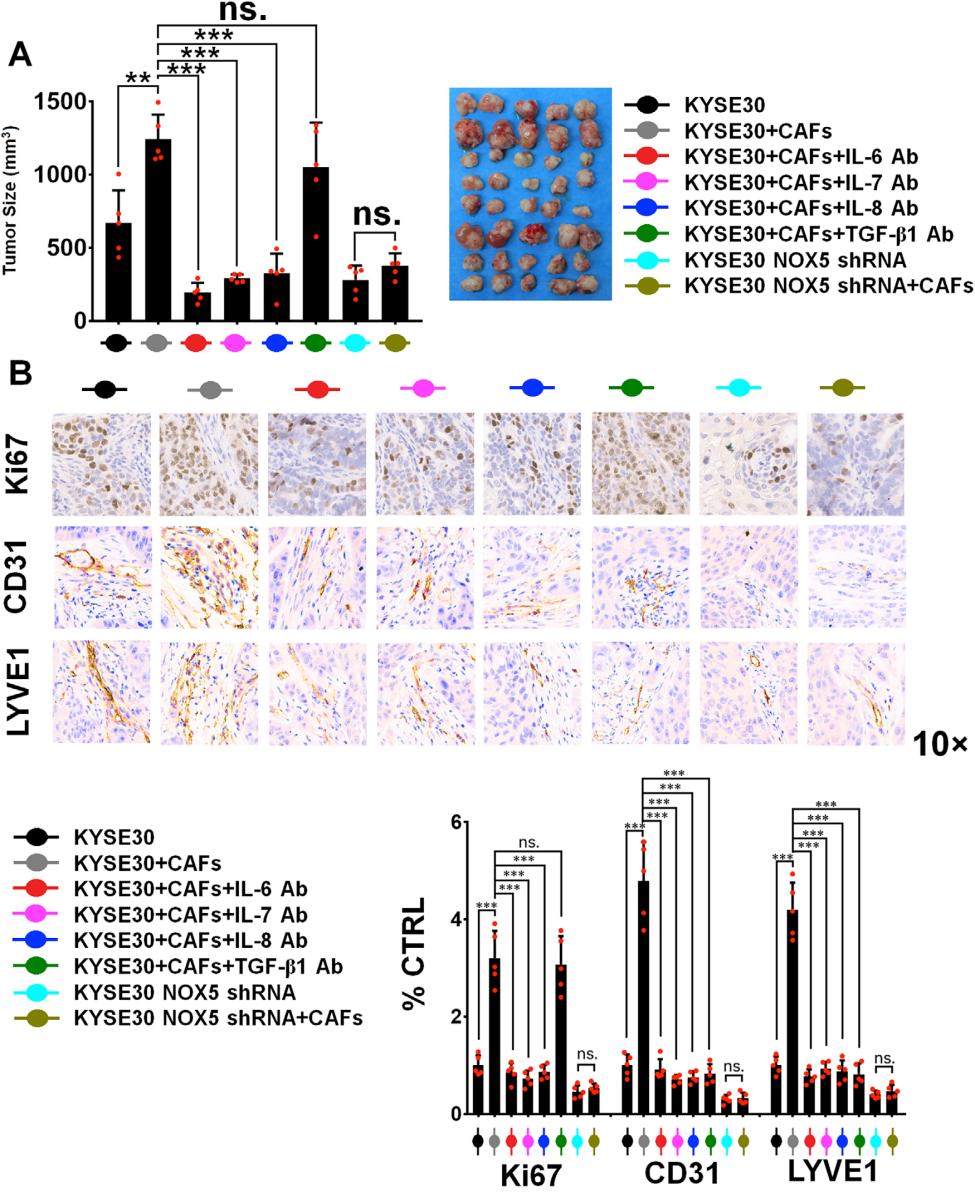
CAFs promote the malignancy of ESCC tumors *in vivo*. Tumor volume was measured at day 33. Mice‐bearing control or NOX5 shRNA KYSE30 tumors with NFs (a mixture of pairs 1, 2, and 3)‐activated CAFs (primed by KYSE30 cells) were treated with control solvent or several Abs, including IL‐6, IL‐7, IL‐8, or TGF‐β1 Ab (10 μg/mouse twice times per week, i.v.). (B) The expression of Ki‐67, CD31, or LYVE1 in indicated KYSE30 tumor tissues was evaluated using IHC assay. n.s. no significant difference; **p < 0.01; ***p < 0.001; two‐tailed unpaired Student's *t*‐test. Error bars, mean ± SD of five independent experiments

**FIGURE 10:**
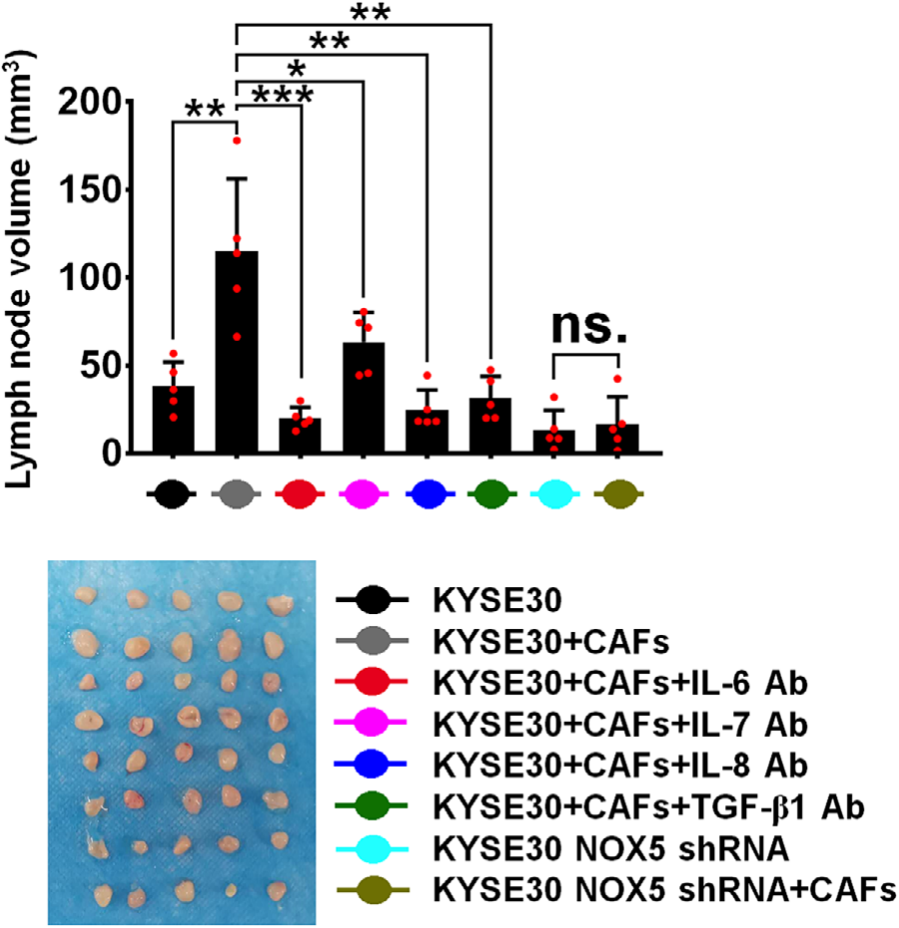
CAFs promote the malignancy of ESCC tumors *in vivo*. A popliteal lymph node metastasis model was established in mice (*n* = 5 biologically independent mouse/group) by inoculating the foot pads with KYSE30 cells and NFs (a mixture of pairs 1, 2, and 3)‐activated CAFs (primed by KYSE30 cells). The popliteal lymph nodes were enucleated and analyzed 5 weeks after inoculation, and the volumes of popliteal lymph nodes were shown. *p < 0.05; **p < 0.01; ***p < 0.001; two‐tailed unpaired Student's *t*‐test. Error bars represent mean ± SD of five independent experiments

To determine whether CAFs contribute to tumor malignant progression *in vivo*, we subcutaneously cotransplanted KYSE30 cells with activated CAFs into female NOD/SCID mice. KYSE30 cells cotransplanting with activated CAFs significantly enhanced tumor volume (Figure 9A), increased the expression of proliferative biomarker‐Ki67, lymphangiogenic biomarker‐LYVE‐1, and microvessel biomarker‐CD31 (Figure 9B), compared with KYSE30 tumors alone. However, activated CAFs were unable to enhance the malignancy of NOX5‐depleted KYSE30 tumors (Figure 9A and B). Furthermore, IL‐6, IL‐7, IL‐8, and TGF‐β1 Abs (10 μg/mouse twice times per week, i.v.) markedly inhibited activated CAFs‐induced KYSE30 tumor malignancy *in vivo* (Figure 9A and B). TGF‐β1 Ab did not have a significant effect on tumor size and Ki67 expression (Figure 9A and B), because TGF‐β may function as tumor proliferation suppressor. Results of Figure S10 showed that the expression of stromal αSMA was significantly upregulated in KYSE30 tumors coinjected with CAFs, compared with KYSE30 tumors alone. The increase expression of αSMA was inhibited by IL‐6, IL‐7, IL‐8, and TGF‐β1 Abs (Figure S10). The effect of CAFs on lymphatic metastasis of ESCC was investigated *in vivo* using a popliteal lymph node metastasis model. The indicated KYSE30 cells and activated CAFs were coinoculated into the foot‐pads of animals. Results of Figure 10 showed that KYSE30 tumors in lymph nodes formed with coinoculated CAFs had larger volumes than those of control KYSE30 tumors alone. However, activated CAFs did not have a significant effect on the volumes of lymph nodes formed by NOX5‐depleted KYSE30 tumors (Figure 10). IL‐6, IL‐7, IL‐8, and TGF‐β1 Abs significantly suppressed the CAFs‐mediated formation of lymph nodes of KYSE30 tumors (Figure 10).

## 4 DISCUSSION

Tumor malignancy is carefully choreographed by the dynamic interplay between tumor cells and stroma.^1^, ^32^, ^33^ Various cytokines and chemokines secreted by tumor cells manipulate TME components from their normal functions to the abnormal tumor‐promoting functions. The production of tumor‐regulatory molecules from activated stroma reciprocally enhances tumor malignancy. However, the detailed mechanisms associated with the interactions between tumor and stroma are still not being unraveled.^5^, ^34^, ^35^ To examine the role of CAFs‐derived cytokines in tumor malignant progression, we distinguished cytokine profiles between NFs and CAFs. The expression of various cytokines, including IL‐6, IL‐7, IL‐8, TGF‐β1, or CCL5, was higher in CAFs than in NFs. Correspondingly, the activity of inflammatory cytokines‐related transcriptional factor‐NF‐κB p65 was more significantly upregulated in CAFs than in NFs and positively correlated with the expression of the abovementioned cytokines. Furthermore, our *in vitro* assays showed that ESCC cells induced a phenotypic conversion of NFs to CAFs and activated the NF‐κB pathway in CAFs to stimulate the secretion of cytokines. Thus, we depicted the cytokine profiles between NFs and CAFs, and further showed the inductive effect of tumor cells on the activation of NFs to CAFs. The cytokine profile of the CAFs in our study is similar to the senescence‐associated secretory phenotype (SASP), which means that senescent cells secrete various cytokines, chemokines, and growth factors to provide these cells with diverse functionalities.^36^, ^37^ Some studies have suggested that the SASP contributes to tumorigenesis by regulating several cellular events, including chromatin remodeling, activation of specific transcription factors, control of mRNA translation, and intracellular trafficking.^36^, ^37^, ^38^, ^39^ Chemotherapeutic agent‐induced DNA damage is the major stress that induces the overproduction of intracellular ROS and activates SASP.^36^, ^37^ Thus, investigating the relationship between SASP, NOX5/ROS axis‐mediated tumor/stroma crosstalk, and chemotherapies will provide applicable evidence for tumor treatment.

The alimentary tract is surrounded by abundant adipose tissue at the adventitia. Adipose tissue‐derived MSCs are one of the most critical sources of CAFs during tumor pathogenesis.^25^ Thus, the interaction between the adipose‐derived MSCs and ESCC cells was also examined in the present study. Our data showed that ESCC cells activated adipose‐derived MSCs to CAFs similarly with NFs, suggesting that ESCC cells can effectively interact with TME, which ultimately mediates tumor progression.

ROS contributes to the development of cytokine‐mediated chronic inflammation‐associated cancer.^40^, ^41^ However, how ROS activates CAFs is still unclear. In this study, we showed that NOX5, a strong ROS producer overexpressed in ESCC cells, effectively induced NFs and adipose‐derived MSCs to obtain the functional and phenotypic properties of CAFs. Mechanistically, our results indicated that NOX5 oxidized and activated Src, which stimulated the tyrosine phosphorylation of IκBα to fully activate NF‐κB in ESCC cells. We hypothesized that after Src oxidization, the formation of intramolecular bond between two cysteine residues is beneficial for Src protein to maintain an activated status, which critically contributes to Src to constantly stimulate the activities of downstream kinase proteins. Our results demonstrated that NOX5 stimulated the intratumoral ROS/Src/NF‐κB/cytokine axis to activate CAFs and further suggested the role of NOX5 as an oncogenic driver for the crosstalk between tumor and stroma.

Cytokines are one of the most critical mediators for the crosstalk between tumor cells and their surrounding TMEs.^42^, ^43^, ^44^, ^45^ Tumor‐associated macrophages (TAMs)‐derived CCL18 activates the FAK or Pyk2/Src signaling to mediate the epithelial‐mesenchymal transition and invasion of breast cancer cells.^29^, ^46^, ^47^ Reciprocally, mesenchymal‐like breast cancer cells secrete GM‐CSF to activate TAMs and then facilitate tumor progression.^29^ In addition to TAMs, activated CAFs, another important TME component, can also affect intratumoral signaling pathways and several malignant phenotypes of tumor cells in a paracrine manner.^48^, ^49^, ^50^ The CAFs‐released cytokines identified in the present study can eventually stimulate the activation of several signaling pathways, especially Stat3 and NF‐κB, in tumor cells.^51^, ^52^, ^53^, ^54^, ^55^ Furthermore, tumor cells‐secreted cytokines can educate stromal fibroblasts.^56^, ^57^ Gastric cancer (GC)‐derived TNF‐α and IL‐1β enhanced the motility of CAFs to induce GC metastases.^13^ Dermal fibroblasts could be activated into CAFs by IL‐1β.^58^ Our results confirm the belief that maintenance of the cytokine loop between tumor and CAFs is essential for tumor metastasis.

Activation of the stromal signaling pathways supports tumor progression.^59^, ^60^ Persistent activation of NF‐κB in the CD10^+^GPR77^+^ CAFs is critical for the paracrine regulation of IL‐6 or IL‐8, which provides a niche for the formation of cancer stem cells.^54^ Activated transcriptional factor‐heat shock factor 1 in CAFs promotes the secretion of TGF‐β and stromal cell‐derived factor 1 to orchestrate the malignancy of tumor cells.^61^ Combined with these reports, our study demonstrates that the activation of NF‐κB/cytokines axis in CAFs is also important for tumor malignancy.

In our study, we found that regulation of the NOX5's expression in ESCC cells effectively modulated lactate secretion. Furthermore, adding lactate to the CM of NOX5‐depleted ESCC cells could effectively restore the ability of tumor cells‐mediated CAFs activation. A previous study identified tumor cells‐derived lactate as an inflammatory stress to drive the NF‐κB/cytokines pathway in endothelial cells and enhance tumor angiogenesis.^62^ Collectively, we provide a novel mechanism that intratumoral ROS links the tumor‐derived metabolite to its surrounding TME.

The generation of new lymphatic vessels through lymphangiogenesis and lymphatic metastasis is considered a critical step in cancer metastasis. Our results showed that activated CAFs also actively induce lymphangiogenesis and lymphatic migration, broadening the function of CAFs in mediating ESCC progression. In addition to the paracrine effect on tumor cells and LECs, the upregulated secretion of stromal cytokines can act in an autocrine manner to sustain the phenotype of CAFs.^12^, ^58^, ^63^ Acquisition of cytokine or chemokine self‐regulatory loop effectively initiates the activation of NFs to CAFs. However, the driving force that triggers the formation of this loop remains unclear. Our data showed the existence of a self‐activated cytokine loop in fibroblasts surrounding ESCC and suggested that this loop is partially initiated by NOX5‐positive ESCC cells.

## 5 CONCLUSION

**FIGURE 11:**
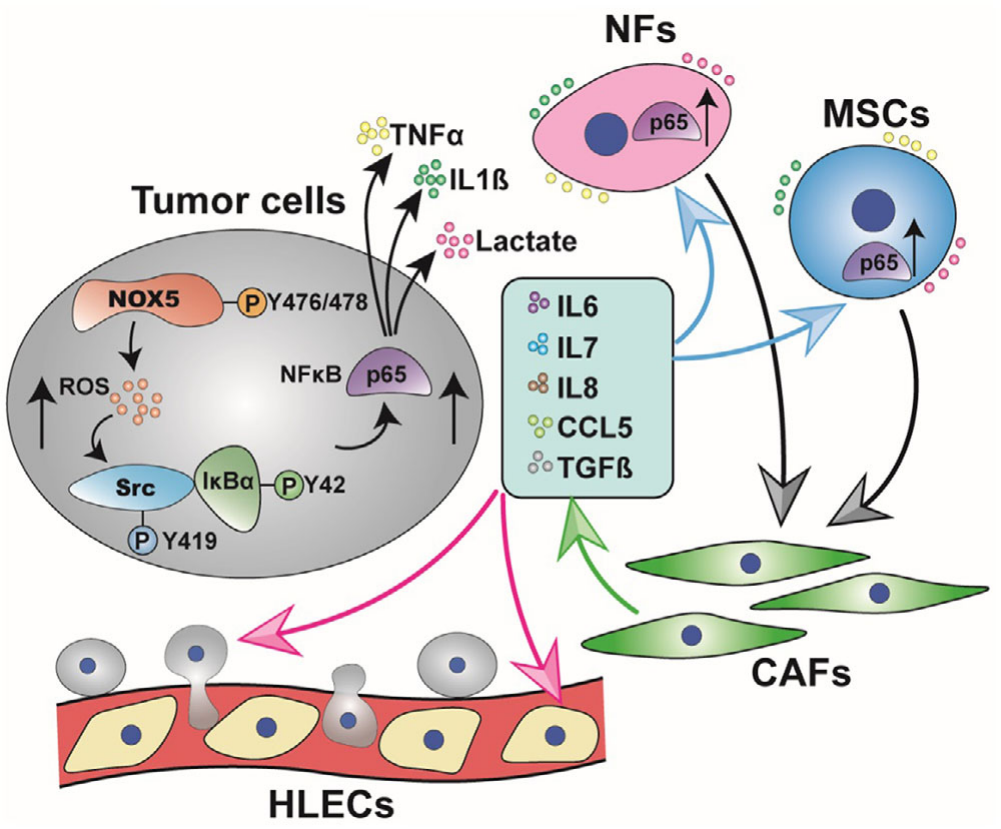
Proposed model of NOX5 activation of CAFs. NOX5 activated Src/NF‐κB signaling to facilitate the secretion of TNF‐α, IL‐1β, and lactate from tumor cells. Subsequently, these cytokines and metabolite acted on NFs and adipose‐derived MSCs, and in turn, stimulated NF‐κB activity in activated CAFs to produce IL‐6, IL‐7, IL‐8, CCL5, and TGF‐β1, which could feedback induce the malignant progression of NOX5‐positive tumor cells

Notably, we concluded that several stromal components, such as fibroblast and adipose tissues, were activated to CAFs and resultantly induced the malignant progression of NOX5‐positive tumor cells. This biological event is largely depended on persistent communication between tumor cells and stroma. Correspondingly, our data provide significance for the understanding of oncogenic driver to form and maintain the cytokines network between tumor cell and its surrounding microenvironment (Figure 11).

## COMPETING INTERESTS

The authors declare that they have no competing interests.

## AUTHOR CONTRIBUTIONS

Q.Z. designed the experiments and wrote the paper. J.C., Y.W., W.Z., D.Z., L.Z., J.Z., and J.F. performed the experiments and analyzed the data.

## Supporting information

Supporting Information

## Data Availability

The data of this research were available from the corresponding author upon reasonable request.
