# Supplementary material for: NOX5 mediates the crosstalk between tumor cells and cancer‐associated fibroblasts via regulating cytokine network
Source: Clin Transl Med. 2021 Aug 3;11(8):e472. doi: 10.1002/ctm2.472 (PMC8329696; doi:10.1002/ctm2.472)
Supplement: Supplementary file 1 — Supporting Information [file CTM2-11-e472-s001.doc]

**Supplementary Figure and Figure Legends**

**
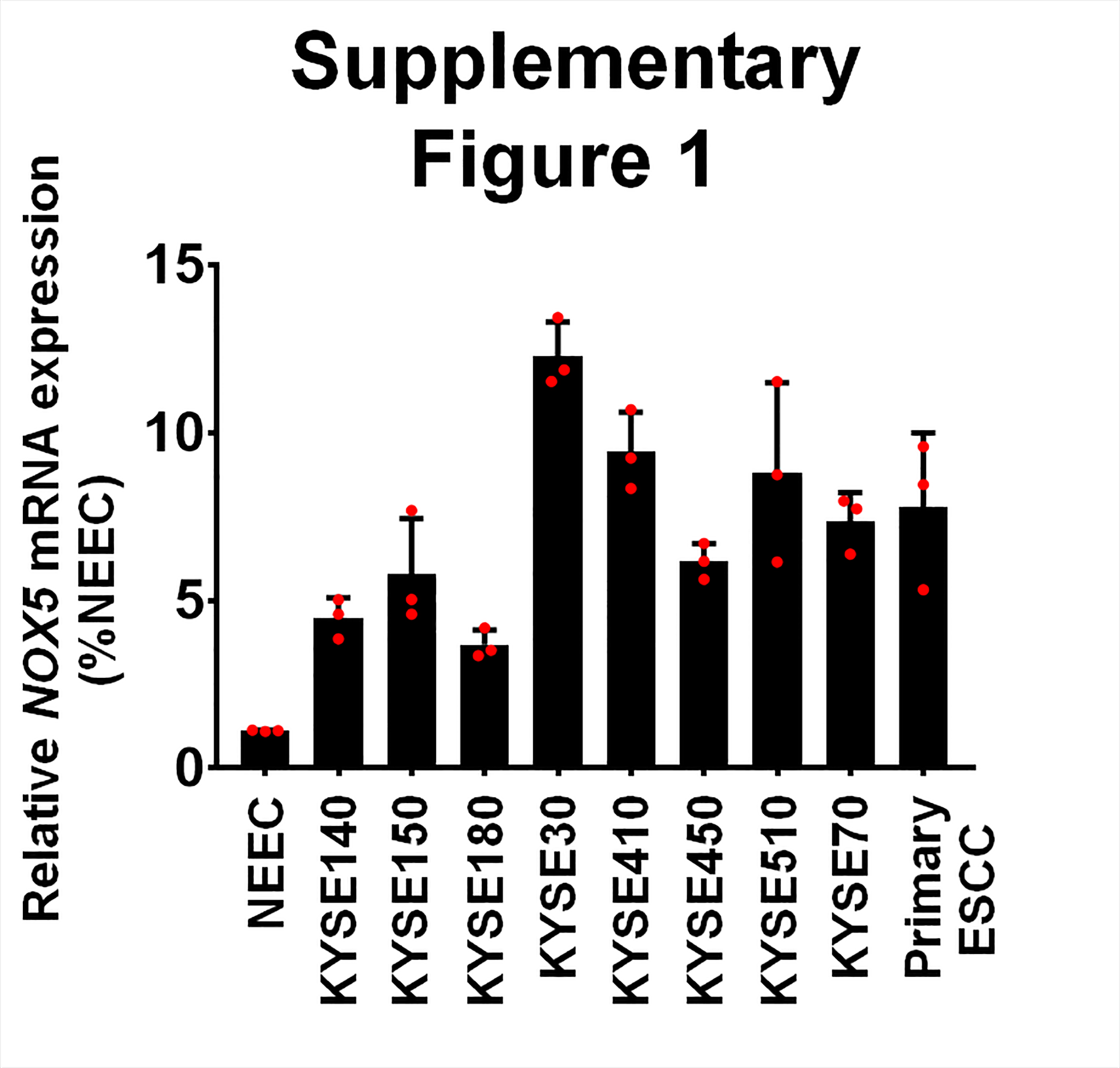
**

**Figure S1. The mRNA level of *NOX5* is upregulated in ESCC cell lines**

PCR analysis of *NOX5* mRNA in 1 primary NEEC, 8 cultured ESCC cell lines, and 1 primary ESCC cells. Error bars, mean ± SD of three independent experiments.


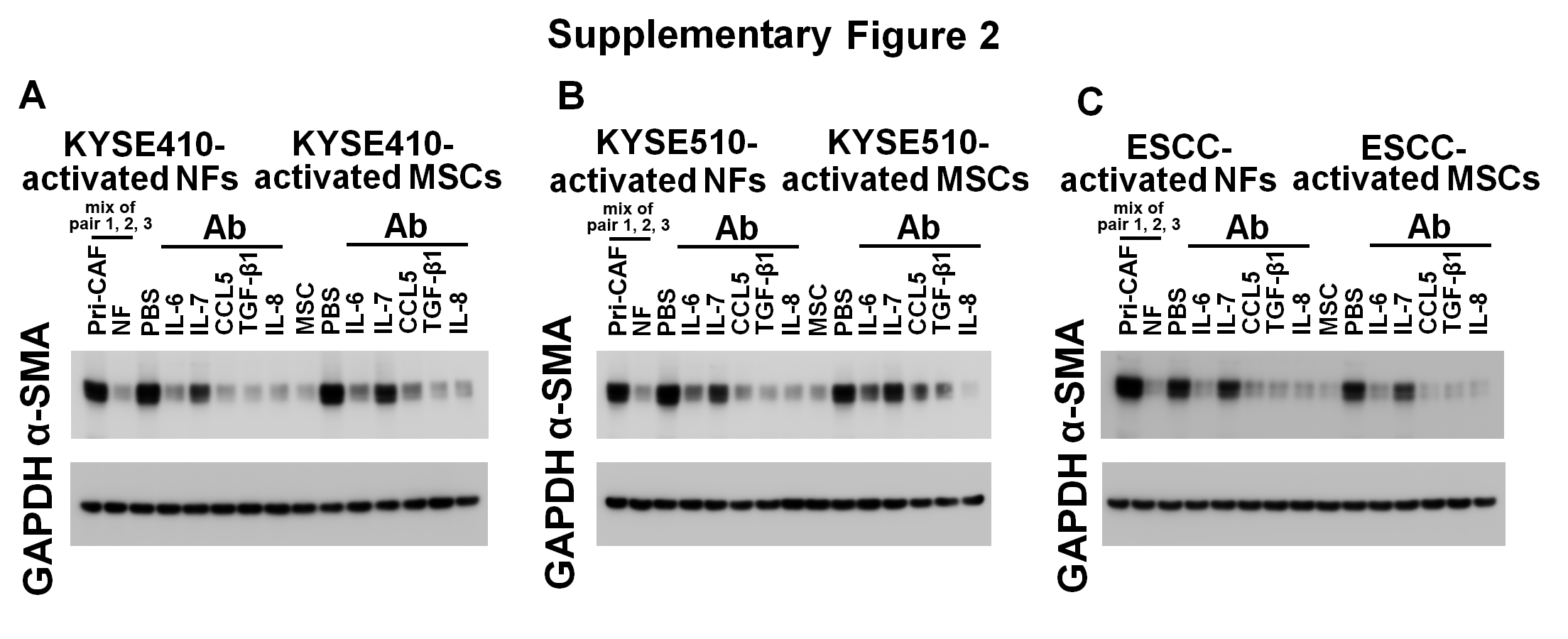


**Figure S2. ESCC cells-activated CAFs induces the activation of NFs and MSCs via secreting of various cytokines**

The experimental condition of Figure S2 was consistent with that of Figure 2. (A-C) NFs (mixture of pair 1, 2, and 3) or adipose-derived MSCs were cultured with the CM from corresponding NFs or adipose-derived MSCs-activated CAFs (primed by KYSE410 (A), KYSE510 (B), or primary ESCC cells (C)) alone or in the presence of several Abs, including IL-6, IL-7, IL-8, CCL5, and TGF-β1 (10 μg/ml). Immunoblotting of αSMA in indicated stromal cells.

**
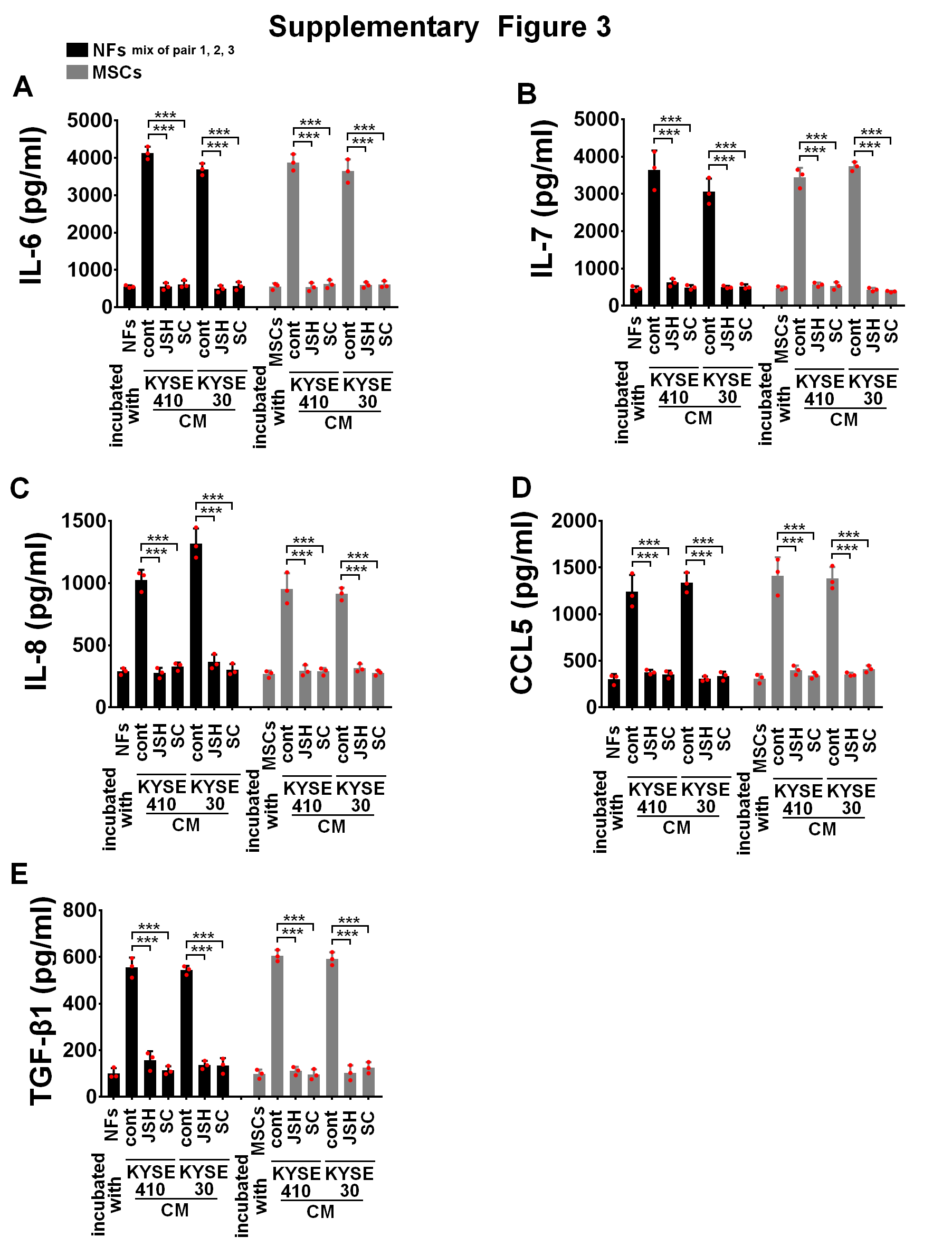
**

**Figure S3. Inhibition of stromal NF-κB activity blocks ESCC cells-induced secretion of cytokines from NFs or adipose-derived MSCs-activated CAFs**

(A-E) NFs (black bars; mixture of pair 1, 2, and 3) or adipose-derived MSCs (gray bars) were incubated with the CM from KYSE30 or KYSE410 cells in the presence or absence of the NF-κB inhibitors (5 μM JSH-23, 5 μM SC75741) for 3 days. Then, tumor CM was removed, fibroblasts were cultured with fresh RPMI1640 medium for 2 days. ELISA assay was used to evaluate the secretion of IL-6 (A), IL-7 (B), IL-8 (C), CCL5 (D), and TGF-β1 (E) from NFs (mixture of pair 1, 2, and 3) or adipose-derived MSCs, or their activated CAFs. *** *P* < 0.001; two-tailed unpaired Student's *t*-test. Error bars, mean ± SD of three independent experiments.

**
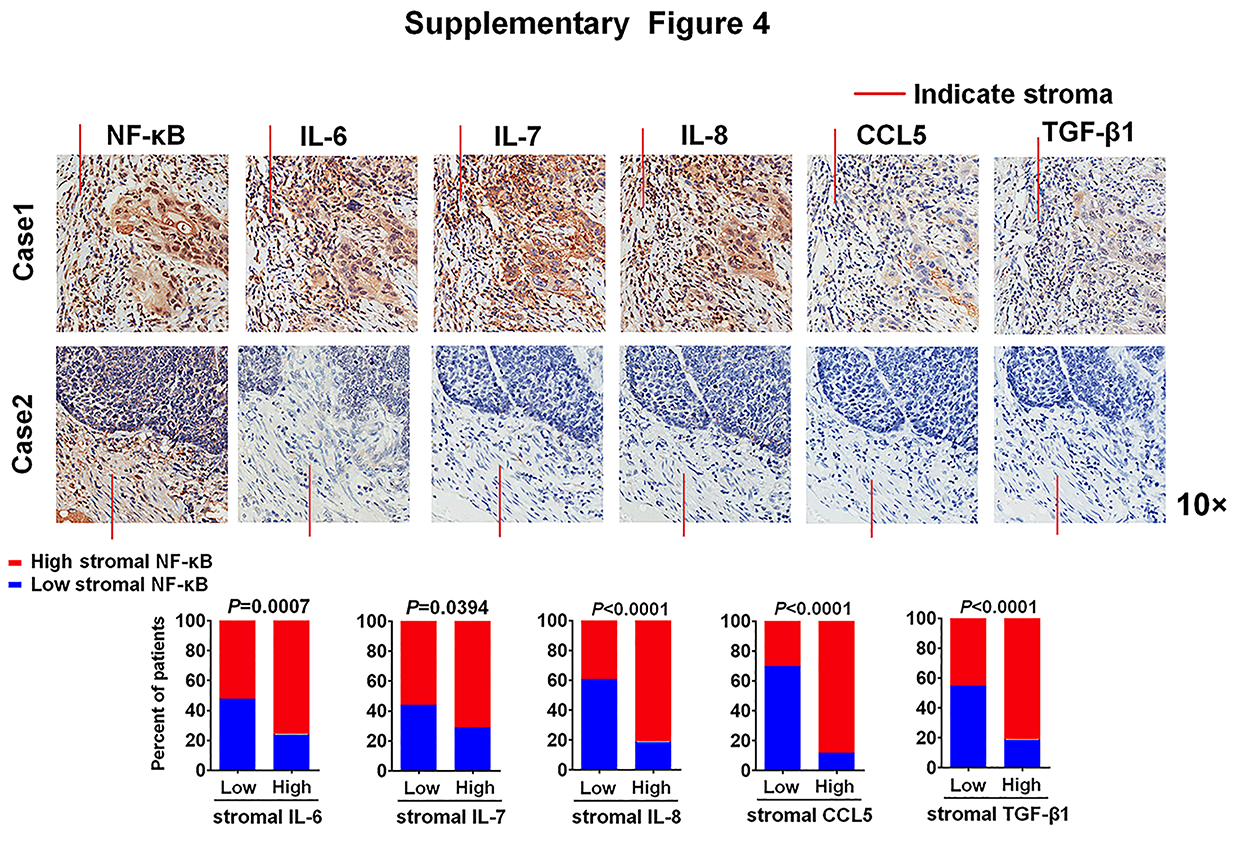
**

**Figure S4. NF-κB is abundantly activated in CAFs and correlated with the levels of cytokines in clinical ESCC samples**

Stromal NF-κB p65 expression was associated with stromal IL-6, IL-7, IL-8, CCL5, and TGF-β1 expression in 54 primary human ESCC specimens. Two representative specimens with low and high levels of Stromal NF-κB expression were shown. Magnification, 10× as indicated. Percentages of specimens showing low or high Stromal NF-κB p65 expression relative to the level of stromal IL-6, IL-7, IL-8, CCL5, and TGF-β1. Two-tailed Pearson χ^2^ test.


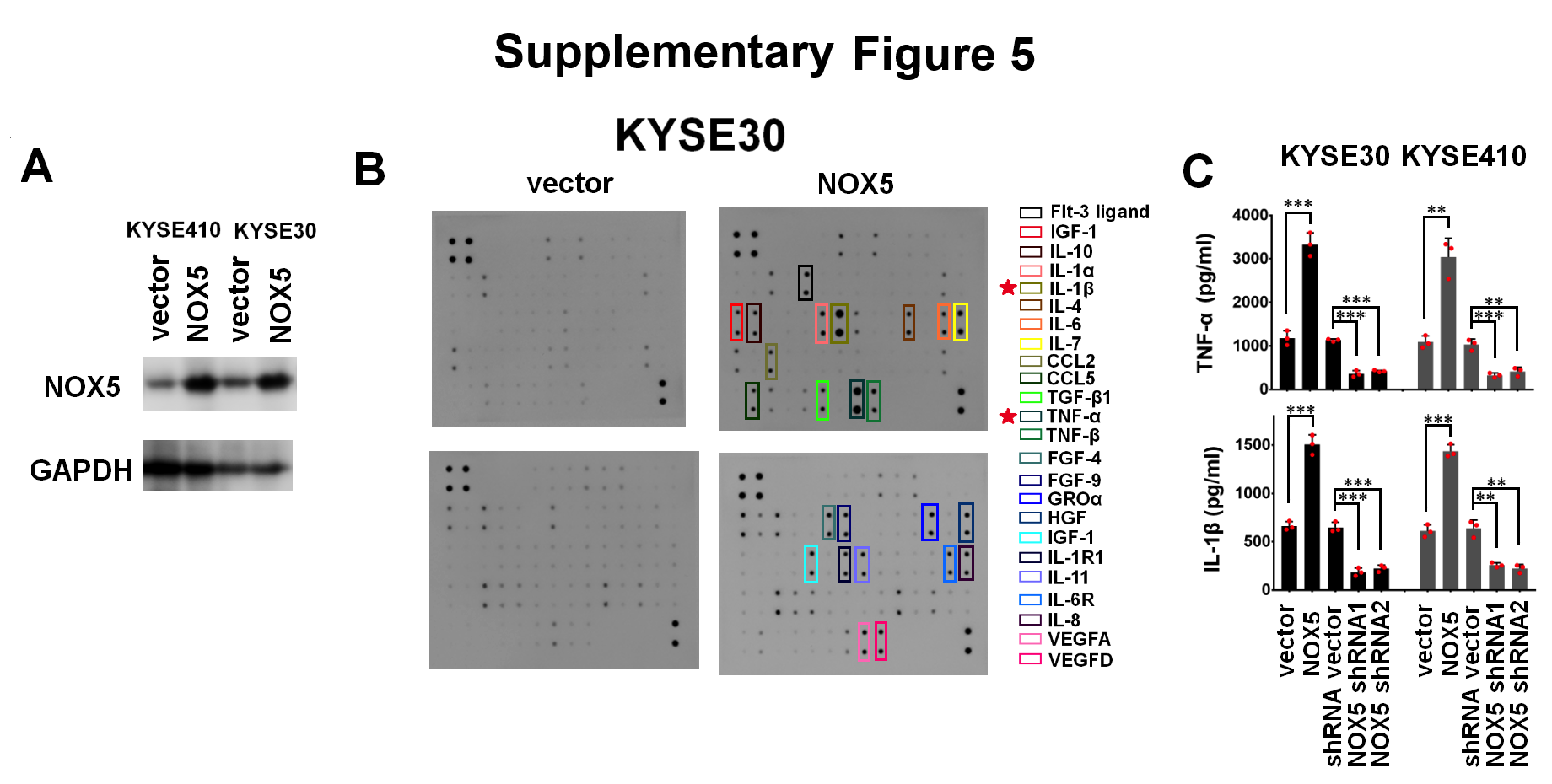


**Figure S5. NOX5 stimulates the secretion of TNF-α or IL-1β from ESCC cells**

1. Stable overexpression of NOX5 in KYSE30 and KYSE410 cells tested by immunoblotting. GAPDH was used as a loading control. (B) CM from the vector control or NOX5-overexpressing KYSE30 cells was analyzed using cytokine antibody array. (C) The concentration of TNF-α and IL-1β in CM from the control or NOX5-overexpressing or shRNA vector or NOX5 shRNA KYSE30 and KYSE410 cells, was assayed by ELISA. ** *P* < 0.01; *** *P* < 0.001; two-tailed unpaired Student's *t*-test. Error bars, mean ± SD of three independent experiments.


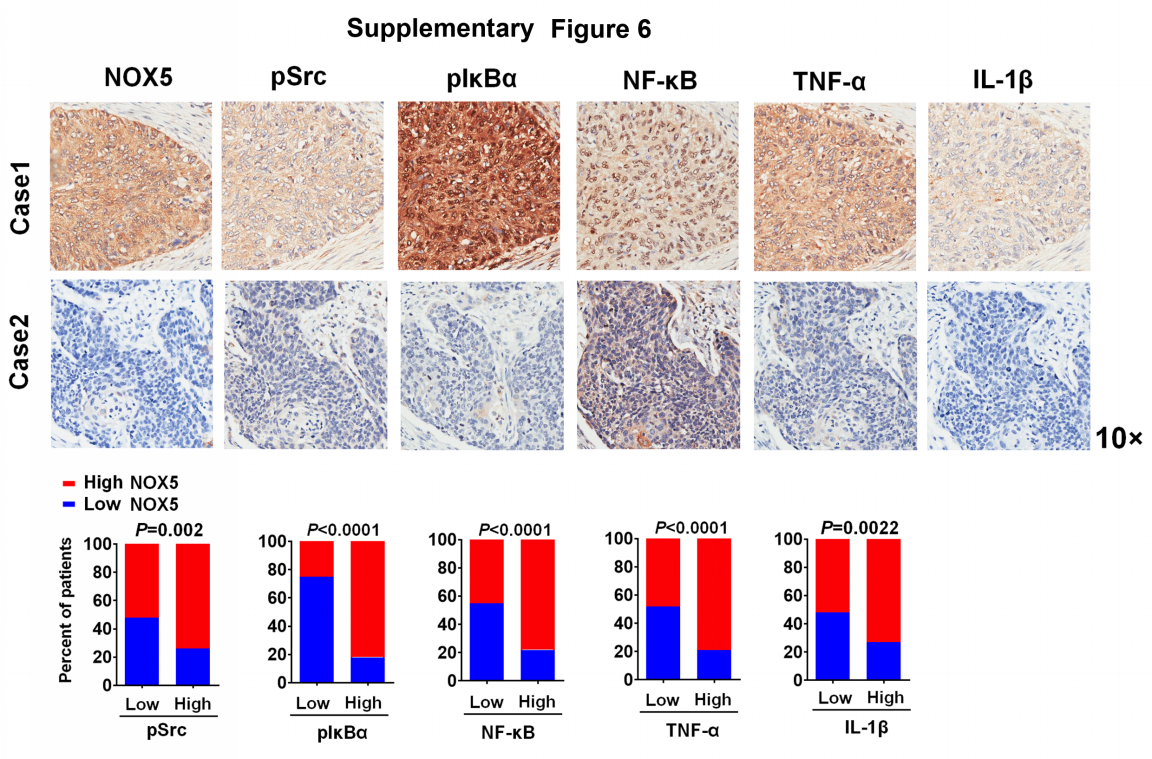


**Figure S6. The positive correlation between NOX5 and Src/ NF-κB/TNF-α/IL-1β signaling in clinical ESCC samples**

NOX5 expression was associated with pSrc, pIκBα, NF-κB p65, TNF-α, or IL-1β expression in 54 primary human ESCC specimens. Two representative specimens with low and high levels of NOX5 were shown. Magnification, 10× as indicated. Percentages of specimens showing low or high NOX5 expression relative to the level of pSrc, pIκBα, NF-κB, TNF-α, or IL-1β. Two-tailed Pearson χ^2^ test.


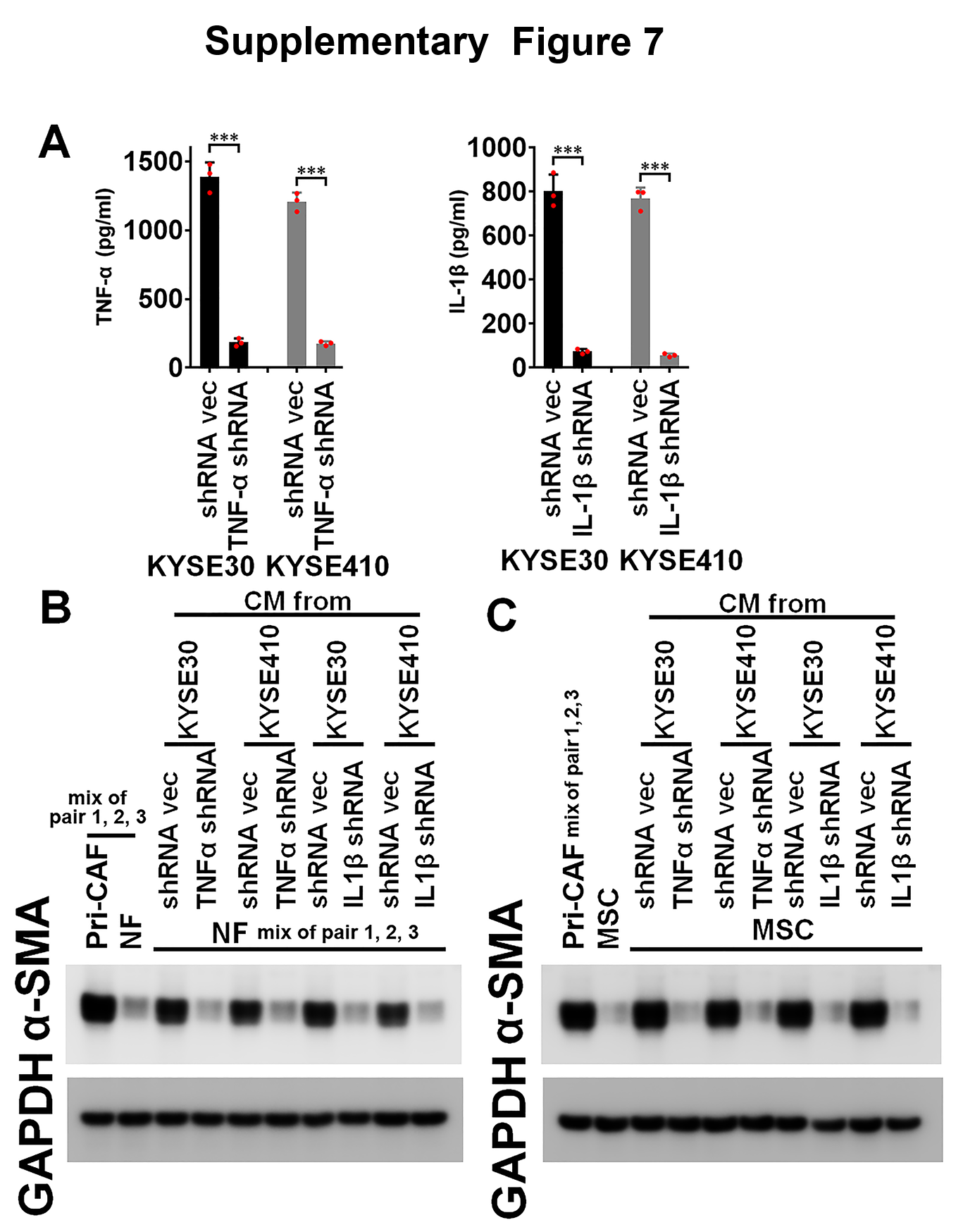


**Figure S7. ESCC cells-secreted TNF-α or IL-1β contributes to CAFs activation**

1. Transient transfection of TNF-α and IL-1β shRNA into KYSE30 and KYSE410 cells. The transfection efficiency was evaluated using ELISA assay. (B-C) NFs (B; mixture of pair 1, 2, and 3) or adipose-derived MSCs (C) were incubated with the CM from KYSE30 or KYSE410 cells harbored control, TNF-α or IL-1β shRNA for 3 days. Then, fibroblasts were cultured with fresh RPMI1640 medium for 2 days. The expression of αSMA was evaluated using immunoblotting. Primary CAFs (mixture of pair 1, 2, and 3) were used as positive control. *** *P* < 0.001; two-tailed unpaired Student's *t*-test. Error bars, mean ± SD of three independent experiments.

**
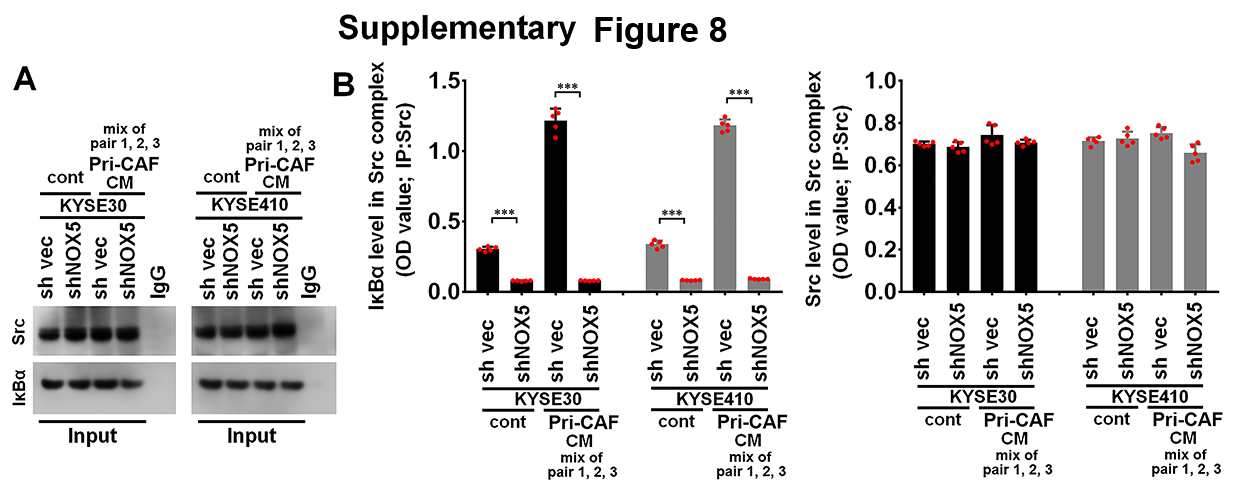
**

**Figure S8. NOX5 promotes the interaction between Src and IκBα**

1. The input and IgG groups of Figure 7B. (B) Src-IκBα ELISA binding assay was applied to quantify the level of IκBα in Src complex (IP: Src) of KYSE30 and KYSE410 control or NOX5 shRNA cells cultured with or without the CM from primary CAFs (mixture of pair 1, 2, and 3). *** *P* < 0.001; two-tailed unpaired Student's *t*-test. Error bars, mean ± SD of five independent experiments.


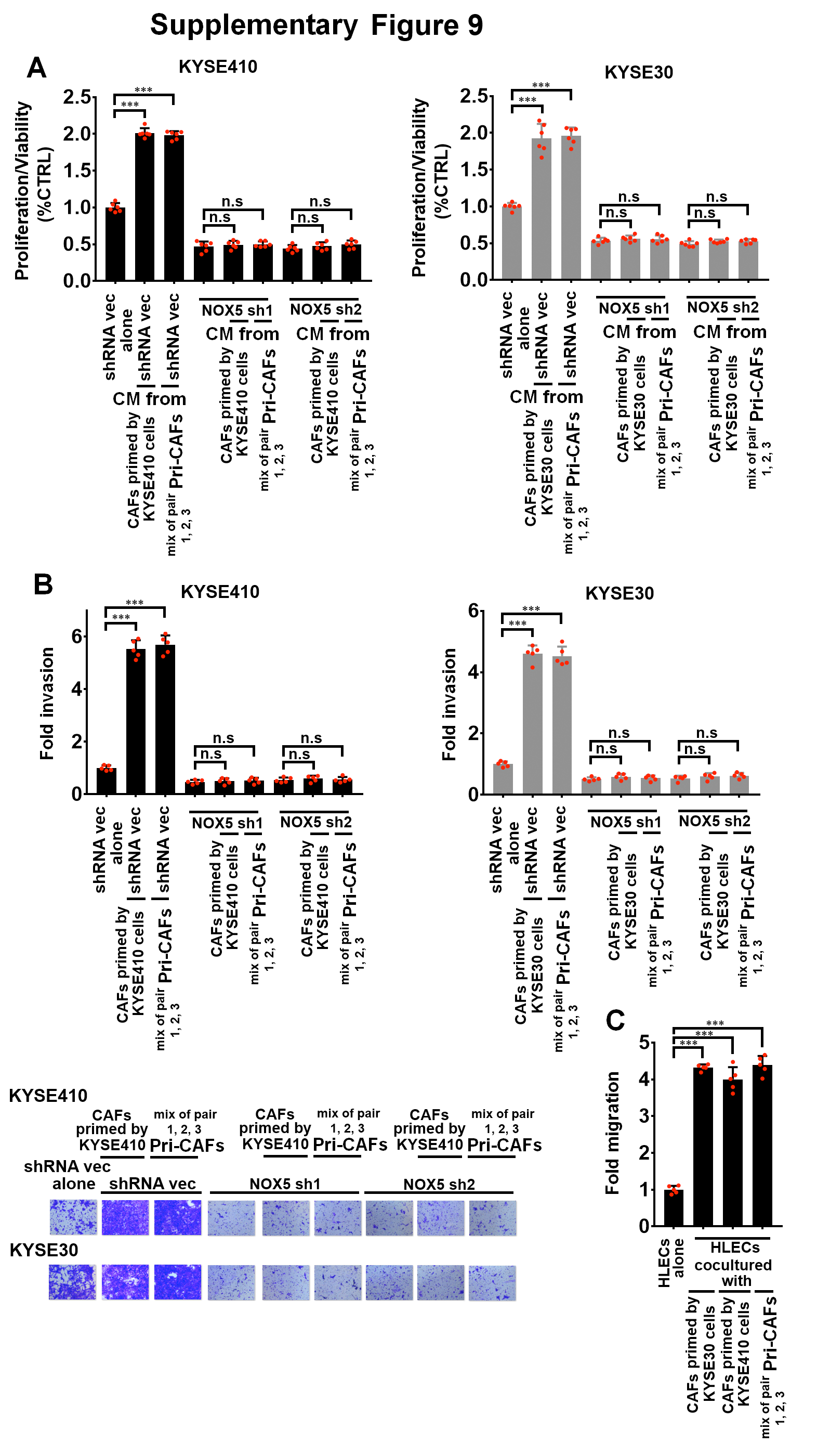


**Figure S9. Primary CAFs induce ESCC malignancy *in vitro***

The experimental conditions of Figure S9 were consistent with those of Figure 8. (A) Growth rates of KYSE30 or KYSE410 cells harbored control or NOX5 shRNA incubated with the CM from corresponding parental KYSE30 or KYSE410 cells-activated CAFs (derive from mixture pair 1, 2, and 3 NFs) or primary CAFs (mixture of pair 1, 2, and 3) for 4 days. Cell growth was assayed by MTS assay. (B) Boyden chamber assay for KYSE30 or KYSE410 cells harbored control or NOX5 shRNA plated on the upper cell culture inserts with their corresponding parental ESCC cells-activated CAFs (derive from mixture pair 1, 2, and 3 NFs) or primary CAFs (mixture of pair 1, 2, and 3) in lower chambers. (C) CytoSelect 96-well cell migration assay for HLECs plated on upper cell culture inserts with NFs (mixture of pair 1, 2, and 3)-activated CAFs (primed by KYSE30 or KYSE410 cells) or primary CAFs (mixture of pair 1, 2, and 3) in lower chambers.n.s. no significant difference; *** *P* < 0.001; two-tailed unpaired Student's *t*-test. Error bars, mean ± SD of five to six independent experiments.


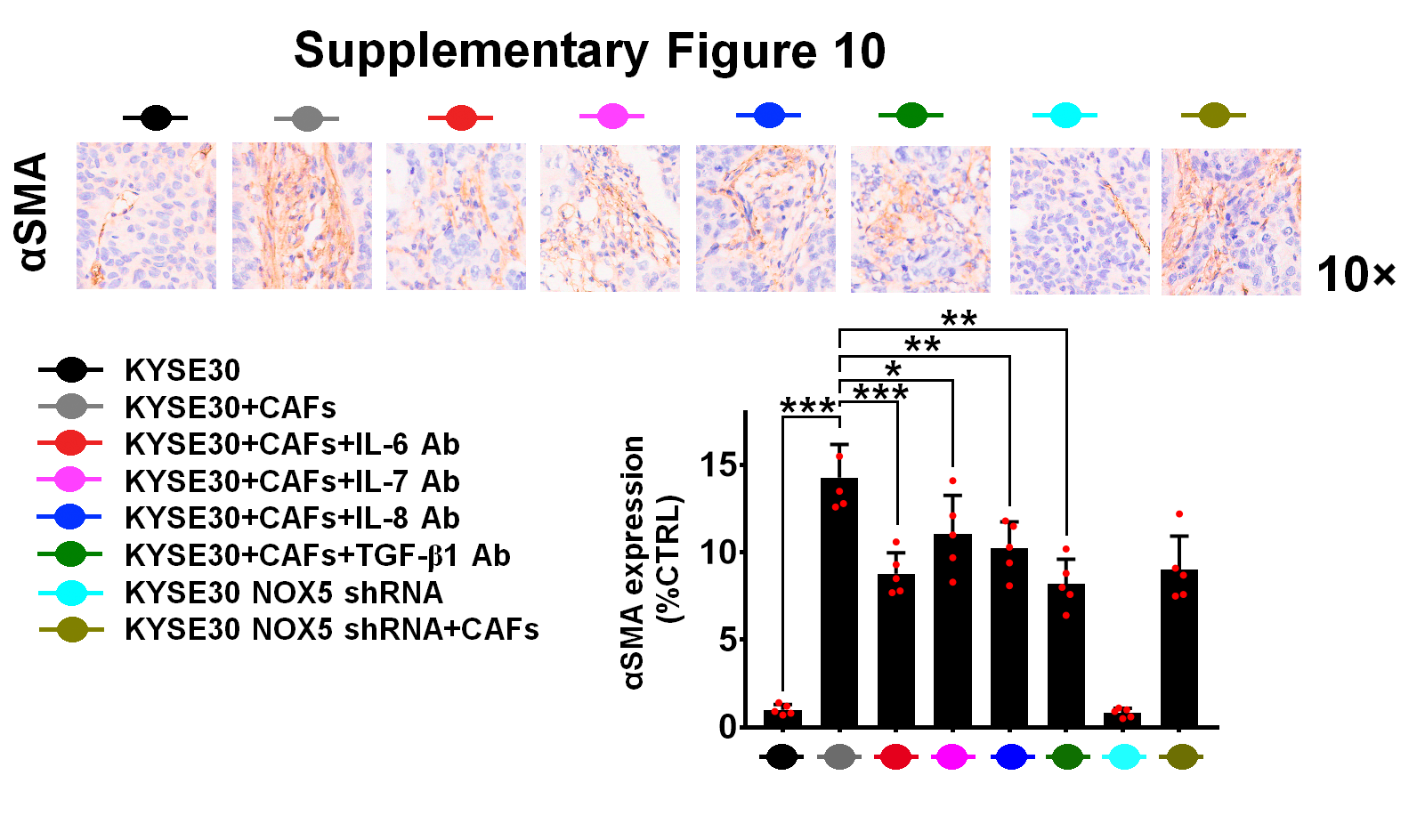


**Supplementary Figure 10. The expression of stromal αSMA in tumor/CAFs coinjection xenografted model**

The expression of stromal αSMA in indicated KYSE30 tumor tissues was evaluated using IHC assay. ** *P* < 0.05; ** *P* < 0.01; *** *P* < 0.001; two-tailed unpaired Student's *t*-test. Error bars, mean ± SD of five independent experiments.

Supplementary Table 1: Information of antibodies and reagents

| **Product Name** | **Catalog Number** | **Company** |
| --- | --- | --- |
| NOX5 antibody | PAB17793 | Abnova |
| IL7 antibody | PAB19553 |
| IL8 antibody | PAB25158 |
| TNF-α antibody, clone CH8810 | MAB6322 |
| IL-1β antibody, clone 3A6 | MAB10697 |
| αSMA antibody, clone ACTA2/791 | MAB14433 |
| Src antibody | 2108 | CST |
| Ki-67 (D2H10) antibody | 9027 |
| CD31 (PECAM-1) (89C2) antibody | 3528 |
| FLAG antibody | 2368 |
| GAPDH (D16H11) antibody | 5174 |
| TGF-β1 antibody | ab215715 | Abcam |
| LYVE1 antibody | ab33682 |
| Phospho-Src-Y419 antibody | AP1027 | Abclonal |
| IκBα Rabbit antibody | A1187 |
| Phospho-IκBα-Y42 antibody | AP0420 |
| Nuclear factor-κB (NF-κB) p65 antibody | SAB4502615 | Sigma-Aldrich |
| catalase-polyethylene glycol | C4963-2MG |
| N-Acetyl-L-cysteine | A9165-5G |
| Src inhibitor dasatinib | S1021 | Selleck Chemicals |
| Src inhibitor PP2 | S7008 |
| NF-κB inhibitor JSH-23 | S7351 |
| NF-κB inhibitor SC75741 | S7273 |
| IL-6 antibody | 119-12232 | Raybiotech |
| CCL5 antibody | 119-15853 |
| Recombinant Human TNF-α Protein | 210-TA | R&D systems |
| Recombinant Human IL-1β Protein | 201-LB |
| Human TNF-α neutralizing antibody | MAB610 |
| Human IL-1β neutralizing antibody | MAB601 |
| Human IL-6 neutralizing antibody | MAB206 |
| Human IL-7 neutralizing antibody | MAB207 |
| Human IL-8 neutralizing antibody | MAB208 |
| Human CCL5 neutralizing antibody | MAB678 |
| Human TGF-β1 neutralizing antibody | MAB240 |

Supplementary Table 2: Characteristics of ESCC cell lines

| ESCC Cell Lines | Characteristics |
| --- | --- |
| KYSE140 | moderately differentiated human squamous cell carcinoma cell line established from esophageal cancer |
|
| KYSE150 | poorly differentiated human squamous cell carcinoma cell line established from esophageal cancer |
|
| KYSE180 | well differentiated human squamous cell carcinoma cell line established from esophageal cancer |
|
| KYSE30 | well differentiated human squamous cell carcinoma cell line established from esophageal cancer |
|
| KYSE410 | poorly differentiated human squamous cell carcinoma cell line established from esophageal cancer |
|
| KYSE450 | well differentiated human squamous cell carcinoma cell line established from esophageal cancer |
|
| KYSE510 | well differentiated human squamous cell carcinoma cell line established from esophageal cancer |
|
| KYSE70 | poorly differentiated human squamous cell carcinoma cell line established from esophageal cancer |
|

| Supplementary Table 3. Cell lines and primary cells used in present study | | |
| --- | --- | --- |
| Cell lines | Number | Origin |
| KYSE140 | 1 | Dr. Yutaka Shimada of Kyoto University |
| KYSE150 | 1 | Dr. Yutaka Shimada of Kyoto University |
| KYSE180 | 1 | Dr. Yutaka Shimada of Kyoto University |
| KYSE30 | 1 | Dr. Yutaka Shimada of Kyoto University |
| KYSE410 | 1 | Dr. Yutaka Shimada of Kyoto University |
| KYSE450 | 1 | Dr. Yutaka Shimada of Kyoto University |
| KYSE510 | 1 | Dr. Yutaka Shimada of Kyoto University |
| KYSE70 | 1 | Dr. Yutaka Shimada of Kyoto University |
| Primary cells | Number | Origin |
| normal esophageal epithelial cells (NEECs) | 1 | CHI Scientific, Inc |
| esophageal squamous cell carcinoma ( ESCC) cells | 1 | CHI Scientific, Inc |
| normal fibroblasts (NFs) | 6 | CHI Scientific, Inc |
| cancer-associated fibroblasts (CAFs ) | 6 | CHI Scientific, Inc |
| adipose-derived mesenchymal stem cells (adipose-derived MSCs) | 1 | Sciencell, Inc |
| human lymphatic endothelial cells（HLECs） | 1 | Sciencell, Inc |
